# Two-Dimensional Nanomaterials With Enzyme-Like Properties for Biomedical Applications

**DOI:** 10.3389/fchem.2020.565940

**Published:** 2020-11-27

**Authors:** Shuangfei Cai, Rong Yang

**Affiliations:** ^1^Chinese Academy of Sciences Key Laboratory for Biomedical Effects of Nanomaterials and Nanosafety, Center of Materials Science and Optoelectronics Engineering, Chinese Academy of Sciences Center for Excellence in Nanoscience, National Center for Nanoscience and Technology, University of Chinese Academy of Sciences, Beijing, China; ^2^Sino-Danish Center for Education and Research, Sino-Danish College, University of Chinese Academy of Sciences, Beijing, China

**Keywords:** two-dimensional, nanomaterials, nanozyme, catalysis, biomedical

## Abstract

Recently, remarkable progress has been made in nanozyme research due to the rapid development of nanomaterials. Two-dimensional nanomaterials such as metal nanosheets, graphene-based materials, transition metal oxides/dichalcogenides, etc., provide enhanced physical and chemical functionality owing to their ultrathin structures, high surface-to-volume ratios, and surface charges. They have also been found to have high catalytic activities in terms of natural enzymes such as peroxidase, oxidase, catalase, and superoxide dismutase. This review provides an overview of the recent progress of nanozymes based on two-dimensional nanomaterials, with an emphasis on their synthetic strategies, hybridization, catalytic properties, and biomedical applications. Finally, the future challenges and prospects for this research are discussed.

## Introduction

Enzymes are catalysts that speed up almost all biochemical reactions in cells. They have some inherent defects that make them unfavorable in large-scale applications. Since the fascinating discovery of enzymatically active Fe_3_O_4_ nanoparticles (NPs) for immunoassay (Gao et al., 2007), the last decade has witnessed great advances in nanozyme research (Wei and Wang, 2013; Huang et al., 2019; Jiang D. W. et al., 2019), deriving from the growth of nanoscience and developments in technology. Various types of catalytic nanomaterials (NMs), primarily zero-dimensional (0D) NPs based on metals [e.g., Pt (Ju and Kim, 2015)], bimetallic compounds [e.g., AuPt (He et al., 2010)], metal oxides [e.g., Co_3_O_4_ (Mu et al., 2013)], and metal chalcogenides [e.g., CuS (He et al., 2012)], have been extensively explored to mimic peroxidases (PODs), oxidases (ODs), catalases (CATs), and superoxide dismutases (SODs) for biomedical applications. Despite much progress in the structural design of 0D nanozymes, there are still several obvious drawbacks. Firstly, most metal-based NPs are often used in a disposable manner, which inevitably causes either economic concerns for the precious metals used or environmental issues due to the pollution and toxicity of these heavy metals (Zhang T. et al., 2014). Secondly, it is known that NPs tend to agglomerate because of huge surface energy, which decreases catalytically active sites, impairing catalytic performance (Yang et al., 2004). Lastly, the inherently imperfect surface accessibility of 0D nanostructures is unfavorable and it is difficult to fully exert the biocatalytic capacity to mimic enzymes (Maromeze et al., 2016). Therefore, researchers should develop nanozymes with novel types of structures and functionalities, a significant research frontier in the nanozyme area.

Two-dimensional (2D) NMs within general sheet-like structures are a newly emerging but very important class of materials. Their lateral dimensions are generally one or several orders of magnitude larger than the thickness, with typical morphologies of nanosheets (NSs), nanoribbons, nanoplates, and nanowalls (Zhang, 2015). Different from their 0D counterparts, 2D NMs with these unique shapes could render large specific surface and distinctive physicochemical attributes, especially in terms of extraordinary surface chemistry, due to the exposure of most of the atoms in 2D NMs on their surface. Initial work about layered materials focused on MoS_2_, dating back nearly half a century (Gan et al., 2017). A surge of interest and studies on 2D NMs started after the discovery of graphene in 2004 (Novoselov et al., 2004). Since then, a host of 2D NMs, including layered double hydroxides (LDHs) (Harvey et al., 2016), transition metal dichalcogenides (TMDs) (Zhu et al., 2013), ultrathin metal NMs (Huang et al., 2011), transition metal oxides (TMOs) (Zhou et al., 2017), Xenes (e.g., black phosphorus) (Khan et al., 2020), metal carbides/nitrides (MXenes) (Jiang C. M. et al., 2019), graphitic carbon nitride (*g*-C_3_N_4_) (Yang et al., 2013), hexagonal boron nitride (*h*-BN) (Chen M. M. et al., 2017), and metal-organic frameworks (MOFs) (Ding et al., 2017), have attracted considerable attention in numerous research fields, such as sensing, catalysis/electrocatalysis, batteries, electronics/optoelectronics, supercapacitors, and biomedical areas.

In terms of mechanical, chemical, and optical attributes, the potential biocompatibility and degradability, 2D NMs have been enthusiastically researched in various biomedical fields including biosensing (Oudeng et al., 2018), antibacterial agents (Lu et al., 2017), bioimaging (Ma D. T. et al., 2020), and cancer therapy (Kong et al., 2017). Also, with a single-atom layer or several-atoms-thick layers, 2D NMs possess the highest specific surface areas among all known materials, thus they have large reservoirs and abundant anchoring sites to load and deliver therapeutic agents (Qian et al., 2017). Moreover, the planar structure endows them with unusual properties including light/ultrasonic/magnetic responses and biological behaviors (e.g., endocytosis, biodistribution, biodegradation, and excretory pathways), which evokes the broad interest in developing 2D NMs as biomaterials (Chimene et al., 2015).

The rapid development of 2D NMs as versatile biomaterials has benefited from significant research progress in graphene, especially its bulk-quantity production and surface functionalization to improve its water solubility (Li et al., 2008; Sun et al., 2008), which paved the way for graphene to be potentially utilized for biomedical purposes. Meanwhile, it should be pointed out that, the success of 0D nanozymes in biomedical applications has promoted the exploration of the enzymatic properties of other nanostructures. As the first example of the employment of 2D NMs to mimic enzymes, the carboxyl-modified graphene oxide (GO) NSs were found to be able to mimic horseradish peroxidase (HRP), which were further developed as a glucose biosensor based on POD-like activity (Song et al., 2010). Later, a large number of 2D NMs with enzymatic activities have been successively reported and become a new type of enzyme-mimic, hereafter referred to as 2D nanozymes.

**Figure 1:**
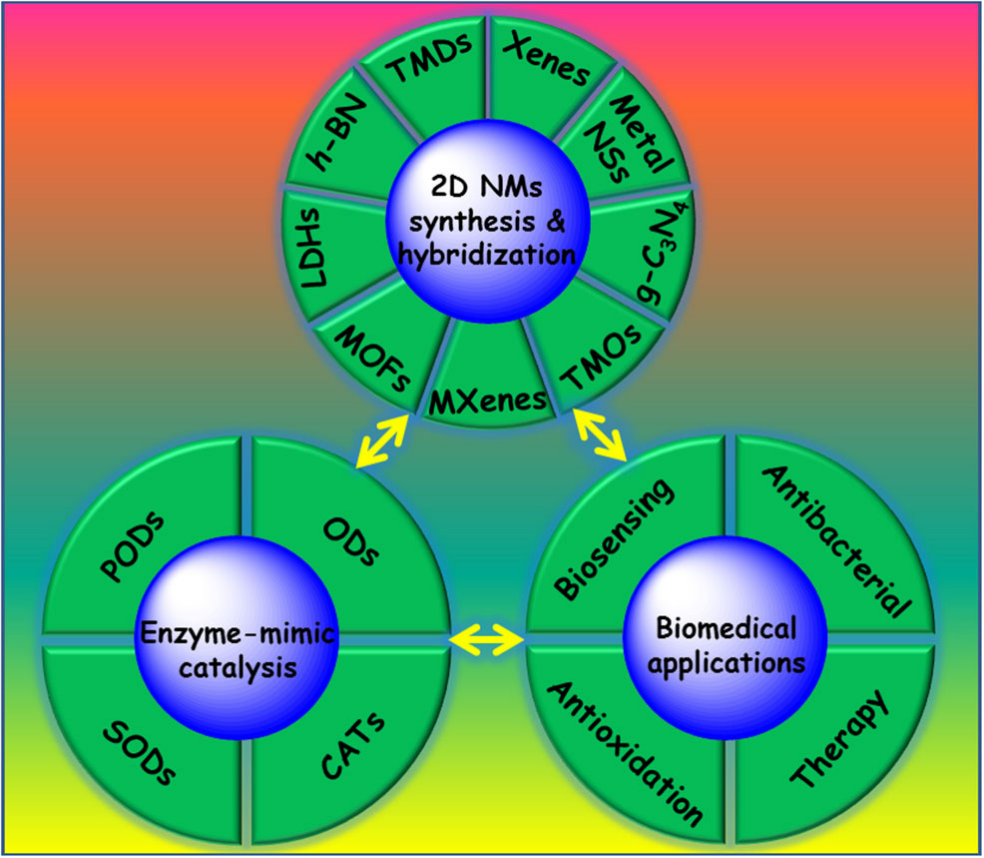
Two-dimensional NMs with enzyme-like activities for biomedical applications.

To our knowledge, despite many reviews (Huang et al., 2016; Agarwal and Chatterjee, 2018; Chen Y. et al., 2018; Dong et al., 2018; Merlo et al., 2018; Tao et al., 2019; Yan et al., 2019; Zhang X. L. et al., 2019; Ren et al., 2020) on the outstanding biomedical performance of 2D NMs, few of them have emphasized enzymatic properties, which are of significant importance to the development of 2D NMs. In this mini-review, we aim to highlight recent progress in 2D NMs with enzymatic properties for biomedical applications. Firstly, we briefly introduce crystal structures, synthesis, and hybridization strategies for 2D NMs. Then, we discuss the enzyme-like activities of 2D NMs. In the following, we summarize the recent advances of 2D nanozymes in diversified biomedical applications, ranging from biosensors, antibacterial agents, and antioxidants to therapeutics (Figure 1). Finally, we share our insights into the development prospects and challenges of 2D nanozymes.

## Crystal Structures, Synthesis, and Hybridization Strategies of 2D NMs

### Crystal Structures of 2D NMs

In essence, the unusual physicochemical attributes of 2D NMs largely depends on their atomic arrangements, which have been clarified in detail elsewhere (Butler et al., 2013; Zhang, 2015; Tan et al., 2017). Despite multifarious composition and structural differences, 2D NMs can be generally categorized as layered and non-layered (Hu et al., 2019). For the former, each in-plane atom connects to the neighboring ones through strong chemical bonding in every layer. The layers, however, stack together via weak van der Waals (VDW) interaction (Bhimanapati et al., 2015). A typical layered compound is graphite, in which each atom covalently bonds to three adjacent atoms in planes by σ-bond but weak VDW interaction exists between layers (Allen et al., 2010). LDHs, TMDs, *g*-C_3_N_4_, *h*-BN, Xenes, MXenes, and MOFs, also have a graphite-like crystallite structure. By contrast, the non-layered NMs crystallize in three dimensions by atomic/chemical bonds to form bulk crystals, which typically include 2D metals, metal oxides/chalcogenides, and others (Tan and Zhang, 2015). Relying on the specific arrangement of atoms, coordination modes between atoms, or stacking order between layers, these non-layered NMs can crystallize into different crystal phases, which greatly affect their attributes and functionalities (Tan et al., 2017).

### Synthetic Approaches of 2D NMs

The top-down and bottom-up methodologies, as two types of commonly used approaches to the synthesis of 2D NMs, are well-summarized in the literature (Chen et al., 2015; Tan et al., 2017). The former is based on direct cleavage of bulk precursors, aiming to break weak VDW interaction between layers in 2D NMs by various driving forces, typically mechanical/liquid-phase/ion-intercalation and exfoliation (Zeng et al., 2011; Huo et al., 2015; Yi and Shen, 2015). Notably, these methods are only suitable for layered materials. They are relatively simple but suffer from certain disadvantages and limitations. Take synthesis of MoS_2_ NSs for example, mechanic exfoliation (Novoselov et al., 2005) is limited by low throughput, thereby making it unsuitable for most biomedical applications: Li-intercalation process not only requires a long time (e.g., 3 days) and high temperature (e.g., 100°C) but results in semiconducting-to-metallic phase-transition of MoS_2_ bulk (Yuwen et al., 2016); liquid exfoliation proposes difficulties in removing high-boiling organic solvents (Coleman et al., 2011).

Contrarily, the bottom-up methods usually begin with small organic or inorganic molecules/atoms, employing crystal growth/assembly into a 2D ordered structure, which includes classical chemical vapor deposition (CVD) (Shi et al., 2010) and wet-chemical synthesis (hydro-/solvo-thermal (Duan et al., 2014; Huang et al., 2014) and the self-assembly (Wu et al., 2015) of crystals, etc.). Since the bottom-up methods are based on the chemical reactions of certain precursors in given synthetic systems, they are more versatile than the top-down methods in enabling access to all types of 2D NMs. Despite the above protocols, it is still challenging to develop an appropriate strategy to synthesize 2D NMs with controlled and desirable structural parameters so as to satisfy the specific requirements.

### Hybridization of 2D NMs

It is known that the hybridization of 2D NMs with functionalized species is an effective strategy to extend and expand their functionalities, which could potentially make them suitable for practical applications. Numerous functionalized species (e.g., atoms, ions, molecules, polymers, and nanostructures), have been modified onto/into 2D NMs by various methods such as doping, adsorption, electrodeposition, covalent functionalization, chemical reduction, and self-assembly (Guan and Han, 2019). For example, to improve water dispersibility and the stability of pristine MXenes NSs in a physiological solution for biomedical applications, Shi's group reported modification of Ti_3_C_2_ NSs with soybean phospholipid by adsorption (Lin et al., 2017). The functionalized materials were found to possess enhanced permeability, stable circulation, and retention ability. In a similar study by Geng's group, titanium carbide NSs terminated with ${\text{Al}(\text{OH})}_{4}^{-}$, obtained by intercalation of Ti_3_AlC_2_ bulk with tetramethylammonium (TMAOH) were modified with polyethylene glycol (PEG) molecules. The functionalized NSs demonstrated excellent stability in various physiological solutions, which were further developed as promising photothermal therapeutic agents (Xuan et al., 2016).

A variety of 0D metal NPs-functionalized 2D NSs have also been reported by different groups, including 0D/2D nanostructured Au/GO (Tao et al., 2013), Au/MOF (Huang et al., 2017a), and Pt/black phosphorus (Ouyang et al., 2018). In recent studies, the *in-situ* growth method was also adopted by different groups for the synthesis of Pt/*h*-BN (Ivanova et al., 2019) and “naked” Au NPs on *g*-C_3_N_4_ NSs (Wu et al., 2019), via the reduction of metal precursors on NSs with various reducing agents like NaBH_4_, ascorbic acid, etc. Our group also constructed a variety of 0D/2D heterostructures, including PtAg NPs-decorated MoS_2_ NSs through the hydrothermal process (Cai et al., 2016), Pt NPs-covered CuO NSs by NaBH_4_ reduction (Wang X. H. et al., 2017), and IrO_2_ NPs-modified GO (Sun et al., 2020) and reduced GO (rGO) (Liu X. L. et al., 2019) NSs by electrostatic adsorption/hydrothermal treatment. The modification of NPs onto 2D NMs could not only prevent NPs from aggregation but inhibit the restacking of NSs, which could facilitate practical applications. However, to access these nanocomposites, a multi-step procedure was generally required for synthesis. In a recent study, our group reported a one-pot fabrication of PtRh NPs-modified Rh NSs (Cai et al., 2019). During the synthesis, Pt atoms/clusters as seeds were first formed by reduction of Pt precursors (H_2_PtCl_6_·6H_2_O), which promoted the reduction of Rh precursors (Rh(acac)_3_) to form PtRh NPs and directed formation of Rh NSs around PtRh NPs. In another work, our group demonstrated the one-pot synthesis of Pd NPs-modified NiCl_2_ NSs, by using a three-step process of “*in situ* reduction-oxidation-assembly” (Cai et al., 2018a). Notably, the pre-preparation and/or functionalization of NSs as well as immobilization of NPs on NSs, generally involved in conventional synthetic protocols, were unnecessary in the above studies.

## Enzymatic Properties of 2D Nanozymes

### POD- and OD-Like Properties

PODs and ODs are a class of known oxidative enzymes in biosystems, which activate H_2_O_2_ and O_2_ to catalyze the oxidation of respective substrates under mild conditions (Wei and Wang, 2013; Huang et al., 2019; Jiang D. W. et al., 2019). To evaluate the POD-/OD-like activity of 2D NMs, catalytic oxidations of enzymatic chromogenic substrates like 3,3',5,5'-tetramethylbenzidine (TMB), which are often carried out in acidic media and produce colored products (**Equation 1**), are chosen as a probe reaction by most researchers (Gao et al., 2007; Song et al., 2010; Tao et al., 2013; Cai et al., 2016, 2018a, 2019; Huang et al., 2017a; Wang X. H. et al., 2017; Ouyang et al., 2018; Ivanova et al., 2019; Liu X. L. et al., 2019; Wu et al., 2019; Sun et al., 2020). The reaction process is tracked by UV-Vis spectroscopy and the color variation of the reaction solution can be easily observed by naked eyes.


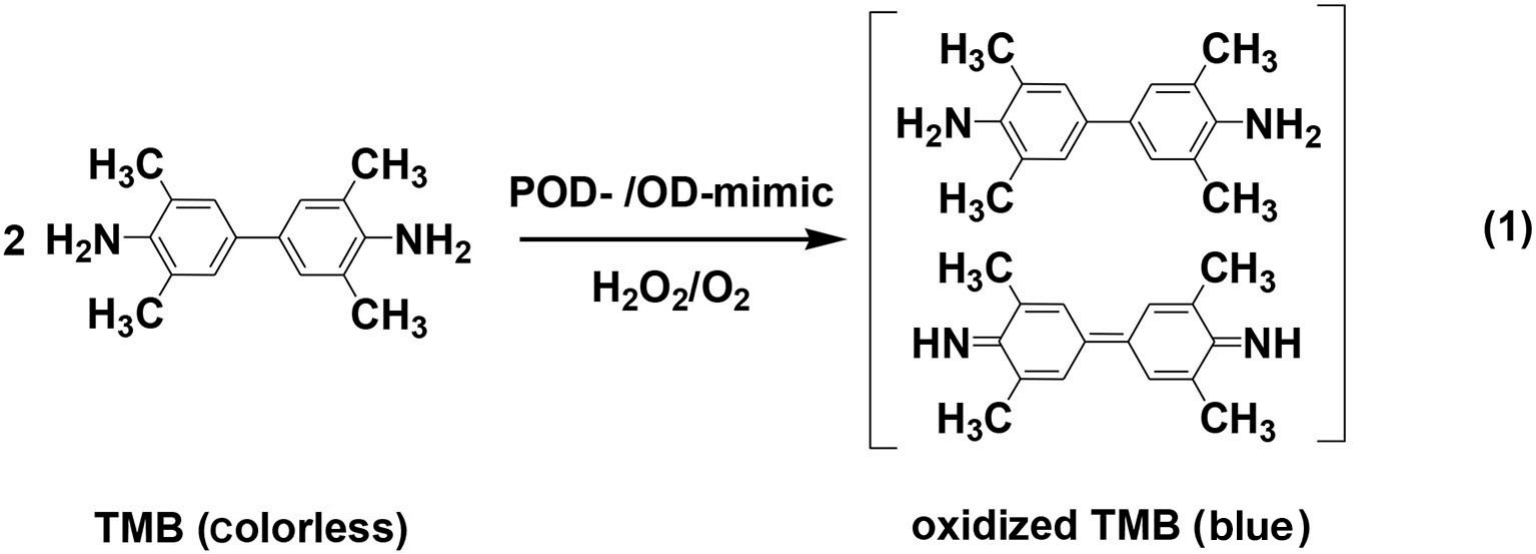


Since the first report of GO NSs as a 2D POD-mimic by Qu's group (Song et al., 2010), similar observations were obtained from other types of 2D NMs, which also exhibited typical Michaelis-Menten kinetics during POD-catalysis. The apparent kinetic constant (*K*_m_) and maximum reaction rate (*V*_max_), as two important parameters related to enzyme-catalysis, could be determined from the Linewearver-Burk plot. The constant *K*_m_ characterizes the binding affinity of a substrate to the enzyme, in which a lower *K*_m_ value means the higher affinity. Table 1 lists the kinetic parameters of typical 2D NMs as POD-mimics based on GO (Zhang L. N. et al., 2014; Sun et al., 2020) or rGO (Liu X. L. et al., 2019), TMDs (Lin et al., 2014a,b; Chen T. M. et al., 2017; Huang et al., 2018; Wu et al., 2018; Feng et al., 2020), LDHs (Zhan et al., 2018; Yang et al., 2020), *g*-C_3_N_4_ (Darabdhara et al., 2019), MOF NSs (Chen J. Y. et al., 2018), *h*-BN (Ivanova et al., 2019), metal oxides (Wang X. H. et al., 2017; Li et al., 2019), and metallic NSs (Wei et al., 2015; Cai et al., 2018b, 2020).

**Table 1:** The kinetics parameters of HRP, Fe_3_O_4_ NPs, and typical 2D POD-mimics^a^.

**Catalyst**	**[E] (10^**−12**^ M)**	**Substrate**	***K*_**m**_ (mM)**	***V*_**max**_ (10^**−8**^ M S^**−1**^)**	***K*_**cat**_ (10^**4**^ s^**−1**^)**	**References**
HRP	25	TMB	0.434	10	0.4	Gao et al., 2007
		H_2_O_2_	3.7	8.71	0.348	
Fe_3_O_4_ NPs	1.14	TMB	0.098	3.44	3.02	Gao et al., 2007
		H_2_O_2_	154	9.78	8.58	
GO	NA	TMB	0.0237 ± 0.001	3.45 ± 0.31	NA	Song et al., 2010
		H_2_O_2_	3.99 ± 0.67	3.85 ± 0.22		
Pt/GO	NA	TMB	0.1864	10.2	NA	Zhang et al., 2014
		H_2_O_2_	221.4	12.45		
IrO_2_/GO	NA	TMB	0.56	32.8	NA	Sun et al., 2020
		H_2_O_2_	5.19	20.8		
IrO_2_/rGO	NA	TMB	0.276	42.7	NA	Liu X. L. et al., 2019
		H_2_O_2_	229	372.9		
MoS_2_	NA	TMB	0.525	5.16	NA	Lin et al., 2014a
		H_2_O_2_	0.0116	4.29		
WS_2_	NA	TMB	1.83	4.31	NA	Lin et al., 2014b
		H_2_O_2_	0.24	4.52		
MoSe_2_	NA	TMB	0.014	0.56	NA	Wu et al., 2018
		H_2_O_2_	0.155	0.99		
WSe_2_	NA	TMB	0.0433	1.43	NA	Chen T. M. et al., 2017
		H_2_O_2_	19.53	2.22		
VS_2_	NA	TMB	0.28	41.6	NA	Huang et al., 2018
		H_2_O_2_	3.49	55.7		
PtAg/MoS_2_	NA	TMB	25.71	7.29	NA	Cai et al., 2016
		H_2_O_2_	0.386	3.22		
N-doped MoS_2_	NA	TMB	0.7916	1.796	NA	Feng et al., 2020
		H_2_O_2_	0.4459	4.348		
NiFe LDHs	NA	TMB	0.5 ± 0.05	NA	NA	Zhan et al., 2018
		H_2_O_2_	2.4 ± 0.1			
CeO_2_/CoFe LDHs	NA	TMB	0.419	NA	NA	Yang et al., 2020
		H_2_O_2_	10.82			
AuNi/*g*-C_3_N_4_	NA	TMB	0.16	2.34	NA	Darabdhara et al., 2019
		H_2_O_2_	4.47	6.16		
Au/*g*-C_3_N_4_	NA	TMB	0.27	1.27	NA	Darabdhara et al., 2019
		H_2_O_2_	11.13	3.44		
Ni/*g*-C_3_N_4_	NA	TMB	0.49	0.75	NA	Darabdhara et al., 2019
		H_2_O_2_	19.91	1.38		
MOF	NA	TMB	0.365	6.53	NA	Chen J. Y. et al., 2018
		H_2_O_2_	2.49	130		
*h*-BN	NA	TMB	0.42	NA	NA	Ivanova et al., 2019
		H_2_O_2_	12.2			
Pt/*h*-BN	NA	TMB	0.21	NA	NA	Ivanova et al., 2019
		H_2_O_2_	9.2			
WO_3_	NA	TMB	10.6	1.53	NA	Li et al., 2019
		H_2_O_2_	1260	3		
Pt/CuO	NA	TMB	0.413	14.6	NA	Wang X. H. et al., 2017
		H_2_O_2_	2.887	8.85		
Pd	NA	TMB	0.21	7.01	NA	Cai et al., 2020
		H_2_O_2_	4.44	4.02		
Pd	5.06	TMB	0.1098	5.82	1.2	Wei et al., 2015
		H_2_O_2_	4.398	6.51	1.3	
Au/Pd	NA	TMB	0.295	19.65	NA	Cai et al., 2020
		H_2_O_2_	5.89	8.19		
Pt/Pd	1.9	TMB	0.0865	6.228	3.1	Wei et al., 2015
		H_2_O_2_	2.231	5	2.5	
Rh	1.53	TMB	0.264	12.56	8.2	Cai et al., 2018b
		H_2_O_2_	4.51	68.09	44.5	

a *K_cat_ = V_max_/E, where E is the concentration of the catalyst*.

*Not available, NA*.

In an earlier study on the POD-like properties of TMDs, Guo's group reported that the commercially obtained MoS_2_ NSs by solution-based exfoliation gave a *K*_m_ value of 0.0116 mM with H_2_O_2_ as substrate, far lower than those of HRP (3.7 mM) (Gao et al., 2007) and Fe_3_O_4_ NPs (154 mM) (Gao et al., 2007), suggesting good affinity of as-obtained materials toward H_2_O_2_ (Lin et al., 2014a). The authors also found the POD-catalysis of MoS_2_ NSs was efficient over a broad pH range (2.0–7.5), wider than those of GO NSs and several 0D NPs [e.g., Fe_3_O_4_ (Gao et al., 2007), Co_3_O_4_ (Mu et al., 2012), and ZnFe_2_O_4_ (Su et al., 2012)]. In a recent study, Wang's group synthesized MoS_2_ NSs using a hydrothermal method, followed by the treatment of N_2_ plasma, producing N-doped MoS_2_ NSs (Feng et al., 2020). With H_2_O_2_ as a substrate, the as-obtained materials not only gave a much lower *K*_m_ value (0.4459 mM) compared to that of the undoped ones (2.0828 mM), but presented a larger *V*_max_ value (4.348 × 10^−8^ M s^−1^) than that of undoped NSs (1.346 × 10^−8^ M s^−1^), suggesting enhanced affinity and the activity of NSs by N-doping. The N_2_ plasma treatment efficiently increased the surface wettability and affinity of pristine NSs, thus improving the access of the electrons and substrates of catalytic reactions. In another study, Das's group synthesized a series of metal NPs (e.g., Au, Ni, and AuNi NPs) decorated *g*-C_3_N_4_ NSs by a solvothermal method (Darabdhara et al., 2019). With a substrate of either TMB or H_2_O_2_, the bimetallic nanocomposites gave both lower *K*_m_ values and larger *V*_max_ values (Table 1) than those of monometallic ones, indicating better affinity and POD-like activity by introducing bimetallic NPs into *g*-C_3_N_4_ NSs. Similar observations were obtained by Pt/CuO (Wang X. H. et al., 2017), IrO_2_/rGO (Liu X. L. et al., 2019), Pd/NiCl_2_ (Cai et al., 2018a), and PtM/MoS_2_ (M = Ag, Cu, and Au) (Cai et al., 2016, 2017; Qi et al., 2016) as reported by our group.

Compared to the layered NM-based POD-mimics, non-layered examples are few. In a study by our group, the Pd NSs were prepared in CH_3_COOH by bubbling of CO gas, followed by Galvanic replacement of Au^3+^ ions to obtain Au NPs decorated Pd NSs (Cai et al., 2020). When TMB and H_2_O_2_ respectively acted as a substrate, the as-obtained nanocomposites afforded two large *V*_max_ values (19.65 × 10^−8^ and 8.19 × 10^−8^ M S^−1^), an ~2-fold enhancement in those of Pd NSs (7.01 × 10^−8^ and 4.02 × 10^−8^ M S^−1^). In another study, Zheng's group synthesized ultrathin Pd NSs with a thickness below 10 atomic layers in the presence of PVP and a halide salt, followed by coating Pt nanodots onto the Pd NSs by reduction of Pt(acac)_2_ with hydrazine hydrate in DMF solution (Wei et al., 2015). As listed in Table 1, compared to Pd NSs, the as-prepared nanocomposites gave lower *K*_m_ values and larger *V*_max_ values, demonstrating superior affinity and activity. To further make a meaningful comparison of catalytic efficiency between Pd NSs and the nanocomposites, the catalytic rate constant (*K*_cat_) was introduced by the authors, which was calculated from the equation *K*_cat_ = *V*_max_/*E*, where *E* is the concentration of the catalyst. With either TMB or H_2_O_2_ as a substrate, the obtained *K*_cat_ value for the nanocomposites was appropriately two times larger than those of Pd NSs. However, compared to several NPs such as Fe_3_O_4_ NPs (Gao et al., 2007), the catalytic efficiency of the nanocomposites is not high enough. In an interesting study, our group presented superior POD-catalysis of single-layer Rh NSs synthesized via the solvothermal method (Cai et al., 2018b). The obtained *K*_m_ values for Rh NSs that were comparable to those of HRP (Gao et al., 2007), while the *V*_max_ value for Rh with H_2_O_2_ as the substrate was exceptionally larger than those of HRP (Gao et al., 2007), Rh NPs (Choleva et al., 2018), and layered NMs based on GO (Gao et al., 2007), and TMD-based NSs (Lin et al., 2014a,b; Chen T. M. et al., 2017; Wu et al., 2018). Moreover, the obtained *K*_cat_ value for Rh NSs to H_2_O_2_ was 128 times, 323 times, and 34 times larger than those of HRP (Gao et al., 2007), Rh NPs (Choleva et al., 2018), and few-layer Pd NSs (Wei et al., 2015), respectively. This high activity could be attributed to the large number of exposed active Rh atoms that were coordinately unsaturated, which facilitated efficient interaction with reactants during catalysis. The Rh NSs also demonstrated satisfactory chemical/thermal stability.

Previous studies revealed that POD-catalysis generally involves two types of pathways: (1) the generation of reactive oxygen species (ROS) including hydroxyl radicals (·OH) (Song et al., 2010; Lin et al., 2014a; Wei et al., 2015; Wang X. H. et al., 2017; Cai et al., 2018a), and (2) electron-transfer (ET) process (Cai et al., 2018b, 2020). The former is believed to undergo a radical chain mechanism, in which the O-O bonds of H_2_O_2_ molecules are broken to generate ·OH radicals, which subsequently oxidize the substrates. In a representative example, Yang's group found that after the coverage of Au NPs onto the *g*-C_3_N_4_ NSs (Wu et al., 2019), the generation of ·OH radicals remarkably increased during catalysis, evidenced by electron spin resonance (ESR). The increase in ·OH radicals could be attributed to the synergistic effect of Au NPs and *g*-C_3_N_4_ NSs, which enhanced the POD-catalysis. By contrast, for the latter, the 2D NMs mediate ET between the substrates and H_2_O_2_ molecules, instead of ROS generation. Take the Au NPs coated Pd NSs reported by our group (Cai et al., 2020) for example, with the introduction of Au atoms, the electronic structure of Pd NSs was modified, which subsequently caused a change in the catalytic pathway of Pd NSs (i.e., from ·OH generation to rapid ET process).

Different from PODs, ODs catalyze oxidations with O_2_ as the oxygen source (generally in the open air), instead of unstable H_2_O_2_. The potential oxidative damage of biological species by H_2_O_2_ could also be avoided by the use of O_2_. The metal oxide NSs like MnO_2_ NSs (Liu et al., 2017; Yan et al., 2017; Ge et al., 2019) were found to exhibit OD-like activities, with the merits of operational simplicity and fine compatibility. Besides, good stability of MnO_2_ NSs as OD-mimics was also found by several research groups. For example, Dyson's group synthesized the MnO_2_ NSs by exfoliation of bulk δ-MnO_2_ in BSA aqueous solution (Liu et al., 2017). After storage for 3 months, no significant loss in the activity of NSs was observed. Similar findings were obtained from Rh NSs (Cai et al., 2018b) and PtRh/Rh nanocomposites (Cai et al., 2019). In some studies, the OD-catalysis was believed to go through the generation of ROS such as superoxide ions (${\text{O}}_{2}^{\cdot -}$), via cleavage of O-O bonds in O_2_ molecules (Cai et al., 2019; Ge et al., 2019).

Notably, besides temperature and substrate concentration, the POD/OD-like activities of 2D nanozymes greatly depend on system pH (often around 4 is efficient), which is similar to the 0D examples and enzymes. Thus, to facilitate their application in biosystems, it is highly desirable to develop 2D nanozymes with a wide range of pH, especially at neutral pH. In an early study, Qu's group synthesized the lysozyme-stabilized Au clusters on GO NSs (Au/GO), which exhibited good catalytic activity over a broad pH range, even in physiological pH (Tao et al., 2013). Compared to that at pH 3.0, the POD-like activity of Au/GO remained about 82 % at pH 7.0. In a recent study, Kim's group reported the rosette-shaped C_3_N_4_ by the polymerization reaction between cyanuric acid and melamine, followed by calcination (Heo et al., 2020). Due to larger surface area and higher porosity, the activity of as-prepared materials was appropriately 10-fold higher than that of conventional bulk-C_3_N_4_. Interestingly, the maximal activity was shown at pH 8.0, and over 80% of the activity relative to the maximum activity remained across a pH range of 6.0–9.0.

Although numerous 2D nanozymes exhibited high activity, the catalysis is also lacking selectivity, similar to the 0D ones. To this end, Lee's group synthesized the N- and B-codoped rGO NSs, which showed high efficiency in POD-catalysis, nearly 1000-fold higher than that of undoped rGO (Kim et al., 2019). More importantly, no OD-like activity was observed for the as-prepared materials, suggesting high selectivity. The same group also provided another valuable example, in which the Fe-N_4_ single site, resembled the heme cofactor present in HRP, was embedded in graphene to obtain the Fe-N-rGO nanocomposites (Kim et al., 2020). Intriguingly, the nanocomposites not only showed a remarkable enhancement in catalytic efficiency, up to appropriately 700-fold higher than that of undoped rGO but had excellent selectivity toward H_2_O_2_. The single-atom nanozymes (SAzymes), as a very new concept in the nanozyme field (Jiao et al., 2019), could open a window to develop a new type of highly active and selective 2D hybrid nanozymes at the atomic scale.

### SOD- and CAT-Like Properties

Contrary to oxidative biocatalysts, SODs and CATs are a type of antioxidant enzyme, which plays an essential part in maintaining redox balance in living organisms by scavenging excess ROS (Liu Y. et al., 2014). There are three forms of human SODs [i.e., cytosolic CuZn SOD, mitochondrial Mn SOD, and extracellular SOD, of which the first is the most studied (Korschelt et al., 2017)]. In a typical cycle of CuZn SOD (Yu et al., 2014; Korschelt et al., 2017), the metal Cu center shuttles between the Cu^2+^/Cu^+^ redox states (**Equations 2, 3**), catalyzing the disproportionation of ${\text{O}}_{2}^{\cdot -}$ into O_2_ and H_2_O_2_ under neutral conditions (**Equation 4**).

(2) $$\begin{array}{l} {\text{O}}_{2}^{\cdot -}+\text{Cu}\left(\text{II}\right)\to {\text{O}}_{2}+\text{Cu}\left(\text{I}\right) \end{array}$$

(3) $$\begin{array}{l} {\text{O}}_{2}^{\cdot -}+\text{Cu}\left(\text{I}\right)+2{\text{H}}^{+}\to {\text{H}}_{2}{\text{O}}_{2}+\text{Cu}\left(\text{II}\right) \end{array}$$

(4) $$\begin{array}{l} 2{\text{O}}_{2}^{\cdot -}+2{\text{H}}^{+}\to {\text{H}}_{2}{\text{O}}_{2}+{\text{O}}_{2} \end{array}$$

Following a similar mechanism to that described above, several NMs based on V_2_O_5_ nanowires (Vernekar et al., 2014) and CeO_2_ NPs (Korsvik et al., 2007) have displayed intrinsic SOD-like activities. The polyvinylpyrrolidone (PVP)-modified Nb_2_C NSs obtained by liquid-phase exfoliation (Ren et al., 2019) also provide an interesting example of SOD-mimics. By density functional theory (DFT) calculations together with the characterization of active intermediates via X-ray photoelectron spectroscopy (XPS) and X-ray diffraction (XRD) analysis, the authors discovered the SOD-like activity of Nb_2_C NSs originated from the surface oxidation process, in which Nb_2_O_5_ formed as catalytically active species.

CATs accelerate the dismutation of H_2_O_2_ into O_2_ and H_2_O, often in the reaction solutions at a high pH (Wei and Wang, 2013; Huang et al., 2019; Jiang D. W. et al., 2019). Previous studies have revealed that the CAT-like activities of 0D metal oxide NPs originated from ion pairs (catalytic sites in two different oxidation states), such as Co^2+^/Co^3+^ (Mu et al., 2013), Fe^2+^/Fe^3+^ (Lin and Gurol, 1998), and Mn^3+^/Mn^4+^ (Hasan et al., 1999). Take Co_3_O_4_ NPs (Mu et al., 2013) for example, the catalytic mechanism can be described as follows. Under alkaline conditions, it is believed that there is a larger concentration of perhydroxyl anions (OOH^−^) in the reaction system (**Equation 5**) (Mu et al., 2013). Since OOH^−^ radicals are more nucleophilic than H_2_O_2_ molecules, they could readily interact with Co(III) centers (**Equation 6**) to generate Co(II) species and release ·OOH radicals (**Equation 7**), while H_2_O_2_ molecules were activated by Co(II) center to produce ·OH radicals (**Equation 8**). The coupling reaction of the generated ·OH and ·OOH radicals gave water and oxygen as final products (**Equation 9**).

(5) $$\begin{array}{l} {\text{H}}_{2}{\text{O}}_{2}+\text{O}{\text{H}}^{-}\to \text{OO}{\text{H}}^{-}+{\text{H}}_{2}\text{O} \end{array}$$

(6) $$\begin{array}{l} \text{Co}\left(\text{III}\right)+\text{OO}{\text{H}}^{-}\to \text{Co}\left(\text{II}\right)\cdot \text{OOH} \end{array}$$

(7) $$\begin{array}{l} \text{Co}\left(\text{II}\right)\cdot \text{OOH}\to \text{Co}\left(\text{II}\right)+\cdot \text{OOH} \end{array}$$

(8) $$\begin{array}{l} \text{Co}\left(\text{II}\right)+{\text{H}}_{2}{\text{O}}_{2}\to \text{Co}\left(\text{III}\right)+\cdot \text{OH}+\text{O}{\text{H}}^{-} \end{array}$$

(9) $$\begin{array}{l} \cdot \text{OH}+\cdot \text{OOH}\to {\text{H}}_{2}\text{O}+{\text{O}}_{2} \end{array}$$

The above mechanism could also apply to 2D counterparts. For example, Jiang's group prepared MnFe LDHs by simple co-precipitation, which exhibited intrinsic CAT-like activities (Ruan et al., 2018). The authors explained that both the Mn^3+/4+^ and Fe^3+^ species exhibited catalytic activity toward H_2_O_2_, which could be used for H_2_O_2_ decomposition to generate oxygen in cancer tissues, to enhance the effect of oxygen-dependent photodynamic therapy (PDT). In another study, besides the metal center shuttling during catalysis, Zheng's group demonstrated the role of ligands in the CAT-like activity of MOF NSs. The Cu MOF NSs were synthesized based on the coordination reaction between the Cu^2+^ ions and isophthalic acids as ligands, with different substituent groups (e.g., -CH_3_, -NO_2_, -OH, and –NH_2_) at 5-position (Wang et al., 2019). The authors found that the CAT-like activity of as-obtained materials was related to the charge density around Cu atoms in the NSs, in which the nitro-functionalized NSs exhibited the highest activity. Since the nitro group is an electron-drawing group while other groups are electron-donating ones, the positive charge density around Cu atoms for the nitro-modified NSs was the highest, which was favorable for the binding of H_2_O_2_ as an electron donor.

It is also of note that several 2D NMs were found to have multiple enzymatic activities. For example, Yang's group found that the few-layer MoS_2_ NSs prepared by liquid-exfoliation exhibited ternary activities (POD-, OD-, and CAT-like activities) (Chen T. M. et al., 2018). As discussed above, these enzymatic activities could be closely associated with specific reaction conditions especially system pH, in which the acidic conditions are usually favorable for POD/OD-mimic catalysis (Qi et al., 2016; Cai et al., 2017; Darabdhara et al., 2019; Feng et al., 2020), while a high pH is beneficial to CAT-catalysis (Liu Y. et al., 2014; Ruan et al., 2018; Wang et al., 2019). Due to the use of various 2D NMs in biomedical fields, for a meaningful discussion of enzyme-like activity, the reaction parameters, nature of materials (e.g., morphology, structure, composition, and size), capping agents, and surface charge, are believed to be taken into account (Cai and Yang, 2020).

## Biomedical Applications of 2D Nanozymes

### Toxicology of 2D NMs

To access the full potential of 2D NMs for practical applications, it is necessary to know their toxicity, including *in vitro* cellular uptake, location, toxicity, *in vivo* biodistribution, degradation, and excretion. However, to date, few studies have highlighted biocompatibility.

The *in vitro* biocompatibility of graphene-based materials, as the oldest and most studied 2D examples, was found to be highly related to their structural/compositional parameters and physicochemical properties, such as morphology, size, layer numbers, hydrophobicity/hydrophilicity, dispersion, and concentration (Ghosal and Sarkar, 2018). For example, hydrophobic and large-size graphene had higher cytotoxicity than hydrophilic and nanosized counterparts (Sukumar et al., 2020). Cui's group found that the cytotoxicity of GO was affected by their concentration, in which the presence of GO with a low concentration (<10 μg mL^−1^) slightly decreased the viability of human fibroblast cells (<20% when exposed for 4 days), but high concentrations of graphene (>50μg mL^−1^) showed obvious cytotoxicity even after only 1 day of exposure (>20%) (Wang et al., 2011). This was caused by the agglomerates of the physiological medium formed between GO layers *via* π - π interactions. Since the aggregated macroparticles could not enter into the cells, they became entrapped on the cell membrane and gave rise to cytoskeleton disruption, membrane deformation, and an increase in intercellular stress, ultimately resulting in cell death. In addition, Koyakutty's group observed that the cytotoxicity of pristine graphene could be improved by carboxyl functionalization (Sasidharan et al., 2011). As the concentration of pristine graphene increased from 0 to 300 μg mL^−1^, the viability of Vero cells decreased remarkably. A lower concentration of pristine graphene (100 μg mL^−1^) resulted in the death of appropriately 50% of cells, which further increased to about 60% at 300 μg mL^−1^. Contrarily, the functionalized graphene showed negligible effects on the viability, even at the highest concentration studied. In another study by the same group, functionalized hydrophilic graphene was found to be favorable for macrophage cell (RAW 264.7) uptake (Sasidharan et al., 2012), distinct from the hydrophobic pristine graphene. The pristine graphene was primarily accommodated at the cell surface and induced ROS-mediated apoptosis when the concentration was above 50 μg mL^−1^, which has not been found for functionalized graphene at a higher concentration of 75 μg mL^−1^. Despite enhanced cytocompatibility by surface functionalization, all forms of graphene-based materials could lead to ROS generation in mammalian cells, which should be carefully taken into account for biomedical applications (Liao et al., 2011; Gollavelli and Ling, 2012).

In an earlier study on the *in vivo* biodegradability of graphene, Koyakutty's group revealed that time-bound spectral alternations using confocal Raman imaging, such as the formation of the defective D′ band, widening of D and G bands, and increase in I_D_/I_G_ ratio of pristine graphene, embedded in different organs (e.g., lung, liver, kidney, and spleen of mice) over a time of 8–90 days (Girish et al., 2013). These observations arose from the increase in structural disorders in graphene phagocytosed by macrophages. The most enhanced amount of disorder, which was observed for the spleen bound samples, caused complete amorphization after 90 days of intravenous injection. In another study, after intravenous GO administration, the accumulation of GO was increased largely in the lung and liver for a longer time (Liu et al., 2012). GO NSs accumulated because of the possible instability and nonspecific binding of GO with different proteins. Since the blood initially flowed to the lung, more GO NSs were accumulated in the lung than other organs. Dash's group investigated the effect of GO and rGO on blood platelet functions (Singh et al., 2011). In this study, the GO sheets were found to cause strong aggregatory responses in platelets by activating a family of Src kinases, which further led to the release of calcium from intercellular compartments. Moreover, this study revealed that, due to charge distribution on the surface of GO NSs, the intravenous administration of GO in mice could trigger pulmonary thromboembolism. By contrast, rGO was unable to effectively activate platelets because of reduced charge density on the graphene surface.

The exploration of TMDs in biomedical areas began in 2013–2014. Similar to graphene-based materials, after surface modification, several members of TMDs also showed good *in vitro* biocompatibility. A relevant example is the WS_2_ NSs prepared by Liu's group using the Li ions insertion method (Cheng et al., 2014). Without surface modification, the authors found that the WS_2_ NSs exhibited obvious toxicity toward 4T1 (murine breast cancer cells), HeLa (human epithelial carcinoma cells), and 293T (human embryo kidney cells) after incubation for 24 h. At a high concentration of 0.1 mg mL^−1^ of WS_2_ NSs, only about 50% of cells were alive; however, after modification with PEG, the as-obtained NSs demonstrated no significant cytotoxicity under the same conditions. Contrarily, without surface functionalization, Zhao's group prepared the WS_2_ NSs by H_2_SO_4_ intercalation and exfoliation in an aqueous solution, which showed low toxicity toward HeLa cells (Yong et al., 2014). The cell viability remained high (above 85%) even at a high concentration (0.2 mg mL^−1^). The authors outlined that the biocompatibility of unmodified WS_2_ NSs could be attributed to a mild aqueous phase in synthesis, in which the toxic organic solvents/chemicals were unnecessary. Using a similar method, Zhao's group prepared MoS_2_ NSs, followed by coating with chitosan (Yin et al., 2014). Even at a high concentration up to 0.4 mg mL^−1^, the as-obtained NSs demonstrated low cytotoxicity against KB (human epithelial carcinoma cell line) and Panc-1 (pancreatic carcinoma, epithelial-like cell line). The functionalized NSs also showed negligible hemolysis of red blood cells (RBCs), suggesting good blood compatibility. The above studies revealed that the *in vitro* toxicity of TMDs could be affected by synthetic methodology, surface chemistry, and specific cells. For the investigations on *in vivo* biocompatibility, Liu's group evaluated the toxicity of PEGylated WS_2_ NSs (Cheng et al., 2014) toward Balb/c mice, by hematoxylin and eosin (H&E) assay, serum biochemistry assay, and complete blood panel test. In their study, no obvious abnormal behavior of mice at the dose of 20 mg kg^−1^ was observed during the assay (45 days after photothermal therapy). In a similar study by the same group, the PEGylated MoS_2_ NSs also demonstrated no obvious toxicity against Balb/c mice, at a relatively lower dose of 3.4 mg kg^−1^ (Liu T. et al., 2014). However, to fully understand their potential toxicity/metabolism in longer terms, more studies are needed.

Compared to graphene-based materials and TMDs, the biocompatibility/biosafety of other 2D NMs especially Xenes, MXenes, and ultrathin metallic NSs, is less explored. Considering these structural and compositional differences, their solubility, biodegradation, and biocompatibility could be different from each other. Presently, the evaluations of *in vitro*/*vivo* biocompatibility of these materials are in progress (Wang S. G. et al., 2020). Despite the low toxicities of several types of 2D NMs [e.g., MnO_2_ (Gao et al., 2020), *g*-C_3_N_4_ (Liang et al., 2017), and *h*-BN (Mateti et al., 2018)] exhibited in preliminary investigations, it is too soon to confirm the biosafety of 2D NMs at this stage. Thus, careful systematic tests on their toxicity are required before practical applications can be developed.

### Biomedical Applications of 2D Nanozymes

#### Biosensors

The accurate determination of biologically important analytes is of significance for clinical diagnosis. The colorimetric method, as an appealing one for point-of-care (POC) applications with many merits (e.g., low cost, simplicity, and practicality), has attracted considerable interest in biosensing (Song et al., 2011).

With good stability and adjustable catalytic activities, 2D NMs provide useful platforms for *in vitro* colorimetric detection. Recently, the assays for various bioanalytes like small molecules, cancer cells, and ions, have been proposed based on POD-like activities of 2D NMs (Table 2). As a typical example, the colorimetric assays for glucose have been largely developed based on a host of 2D NMs and their hybrids, including graphene derivatives [e.g., GO (Song et al., 2010), FePd/rGO (Yang et al., 2019)], TMDs [e.g., MoS_2_ (Lin et al., 2014a), WS_2_ (Lin et al., 2014b), WSe_2_ (Chen T. M. et al., 2017), VS_2_ (Huang et al., 2018)] and their hybrids [e.g., PtAg/MoS_2_ (Cai et al., 2016)], LDHs [e.g., NiFe (Zhan et al., 2018)], and their hybrids [e.g., CeO_2_/CoFe (Yang et al., 2020)], *g*-C_3_N_4_ (Lin et al., 2014c) and its hybrids [e.g., Au/*g*-C_3_N_4_ (Wu et al., 2019), Pd/*g*-C_3_N_4_ (Zhang W. C. et al., 2019), Fe/*g*-C_3_N_4_ (Tian et al., 2013), and AuNi/*g*-C_3_N_4_ (Darabdhara et al., 2019)], and metallic NSs-based hybrids [e.g., Au/Pd (Cai et al., 2020) and Pt/Pd (Wei et al., 2015)]. Some of them showed excellent analytic performance (e.g., wide linear detection range, high sensitivity, and selectivity) for glucose detection and demonstrated practicality and superiority for real samples. However, it is noteworthy that these materials could only be employed to mimic PODs and that glucose oxidase (GOx) was required for detection, since it catalyzed the aerobic oxidation of glucose to generate H_2_O_2_. Intriguingly, Zhang's group proposed a non-enzyme colorimetric assay for glucose based on GOx- and the POD-like activities of Au/MOF, which catalyzed cascade reactions for detection (Huang et al., 2017a). The multifunctional enzymatic properties of hybridized 2D NMs provide valuable opportunities to develop advanced biosensors.

**Table 2:** Summary of several typical 2D nanozymes for colorimetric detection of various target analytes.

**Analyte**	**Materials**	**Activity**	**LDR**	**LOD**	**References**
Glucose	GO	POD	1–20 μM	1 μM	Song et al., 2010
Glucose	FePd/rGO	POD	1–200 μM	1.76 μM	Yang et al., 2019
Glucose	MoS_2_	POD	5–150 μM	1.2 μM	Lin et al., 2014a
Glucose	WS_2_	POD	5–300 μM	2.9 μM	Lin et al., 2014b
Glucose	WSe_2_	POD	10–60 μM	10 μM	Chen T. M. et al., 2017
Glucose	VS_2_	POD	5–250 μM	1.5 μM	Huang et al., 2018
Glucose	PtAg/MoS_2_	POD	1–10 μM	0.8 μM	Cai et al., 2016
Glucose	NiFe LDHs	POD	0.05–2.0 mM	23 ± 2 μM	Zhan et al., 2018
Glucose	CeO_2_/CoFe	POD	0.05–2.0 mM	15 μM	Yang et al., 2020
Glucose	*g*-C_3_N_4_	POD	5–100 μM	1.0 μM	Lin et al., 2014c
Glucose	Au/*g*-C_3_N_4_	POD	5–100 μM	1.2 μM	Wu et al., 2019
Glucose	Pd/*g*-C_3_N_4_	POD	50–2,000 μM	50 μM	Zhang W. C. et al., 2019
Glucose	Fe/*g*-C_3_N_4_	POD	0.5–10 μM	0.5 μM	Tian et al., 2013
Glucose	AuNi/*g*-C_3_N_4_	POD	0.5–30 μM	1.7 μM	Darabdhara et al., 2019
Glucose	Au/Pd	POD	5–400 μM	0.85 μM	Cai et al., 2020
Glucose	Pt/Pd	POD	0.1–0.5 mM	NA	Wei et al., 2015
Glucose	Au/MOF	GOx, POD	10–300 μM	8.5 μM	Huang et al., 2017a
AA	Pt/CuO	POD	1 μM−0.6 mM	0.796 μM	Wang X. H. et al., 2017
AA	IrO_2_/GO	POD	5–70 μM	324 nM	Sun et al., 2020
GSH	MnO_2_	OD	NA	300 nM	Liu et al., 2017
GSH	MnO_2_	OD	10 nM−5 μM	5.6 nM	Ge et al., 2019
GSH	IrO_2_/rGO	POD	0.1–50 μM	83 nM	Liu X. L. et al., 2019
Cysteine	IrO_2_/rGO	POD	0.1–50 μM	40 nM	Liu X. L. et al., 2019
Homocysteine	IrO_2_/rGO	POD	0.1–50 μM	57 nM	Liu X. L. et al., 2019
Xanthine	Rh	POD	2–80 μM	0.73 μM	Cai et al., 2018b
Xanthine	WO_3_	POD	25–200 μM	1.24 μM	Li et al., 2019
Xanthine	MoSe_2_	POD	0.01–0.32 mM	1.964 μM	Wu et al., 2018
Dopamine	Pt/BN	POD	2–55 μM	0.76 μM	Ivanova et al., 2019
Dopamine	CuS/rGO	POD	2–100 μM	0.48 μM	Dutta et al., 2015
Cholesterol	CuS/BN	POD	10–100 μM	2.9 μM	Zhang et al., 2017
AChe	MnO_2_	OD	0.1–15 mU mL^−1^	35 μU mL^−1^	Yan et al., 2017
MCF-7 cells	Au/GO	POD	NA	1,000	Tao et al., 2013
MCF-7 cells	PtCu/MoS_2_	OD	NA	300	Qi et al., 2016
MCF-7 cells	Pt/GO	POD	NA	125	Zhang L. N. et al., 2014
S^2−^ ions	MoS_2_/*g*-C_3_N_4_	POD	0.1-10 μM	37 nM	Liu et al., 2020
Fe^2+^ ions	MoS_2_	POD	0.01–0.8 μM	7 nM	Wang et al., 2016
Pb^2+^ ions	WS_2_	POD	5–80 μg L^−1^	4 μg L^−1^	Tang et al., 2020

^a^*Acetylcholinesterase*.

*AChe, Linear detection range; LDR, Limit of detection, LOD*.

By taking advantage of POD-/OD-like activity of 2D NMs, the colorimetric assays for other analytes like AA (Wang X. H. et al., 2017; Sun et al., 2020), biothiols [e.g., GSH (Liu et al., 2017; Ge et al., 2019; Liu X. L. et al., 2019), cysteine (Liu X. L. et al., 2019), and homocysteine (Liu X. L. et al., 2019)], xanthine (Cai et al., 2018b; Wu et al., 2018; Li et al., 2019), dopamine (Dutta et al., 2015; Ivanova et al., 2019), cholesterol (Zhang et al., 2017), acetylcholinesterase (Yan et al., 2017), cancer cells (Tao et al., 2013; Zhang L. N. et al., 2014; Qi et al., 2016), ions [e.g., S^2−^ (Liu et al., 2020), Fe^2+^ (Wang et al., 2016), and Pb^2+^ (Tang et al., 2020)], have also been established by different groups. The colorimetric biosensing based on 2D nanozymes continues to be a rapidly growing field.

#### Antibacterial Agents

The POD-like activities of 2D NMs have gained ever-increasing interest in antibacterial applications. For example, Yang's group prepared MoSe_2_ NSs by exfoliation of bulk MoSe_2_ powder in the aqueous solution of carboxyl-modified silk fibroin under sonication conditions (Huang X.-W. et al., 2017). In their study, a high concentration of H_2_O_2_ (100 mM) alone was required to eliminate most Gram-negative bacteria *E. Coli*, while only 100 μM H_2_O_2_ could display excellent antibacterial activity in the presence of MoSe_2_ NSs (50 μg mL^−1^). The effectiveness of a combination of MoSe_2_ NSs and H_2_O_2_ was further verified for disinfection and healing of Kunming mice with infected skin wounds. The study provided a type of TMD-based antibacterial agents with the usage of low-dose H_2_O_2_, which potentially avoid the harmful side effects of high-dose H_2_O_2_ in traditional medical therapy. Similarly, Yin's group (Ma D. Q. et al., 2020) and Gu's group (Wang T. et al., 2020) respectively reported good antibacterial efficacy of lysozyme-modified MoS_2_ NSs and N-doped MoS_2_ or WS_2_ NSs, against *E. Coli* and Gram-positive *Bacillus subtilis*. The POD-mimics catalyzed H_2_O_2_ decomposition to generate ·OH radicals, which promoted bacteria-infected wound healing. Several 2D hybrid NMs like Au/*g*-C_3_N_4_ (Wang Z. Z. et al., 2017) also showed potential in antibacterial agents.

**Figure 2:**
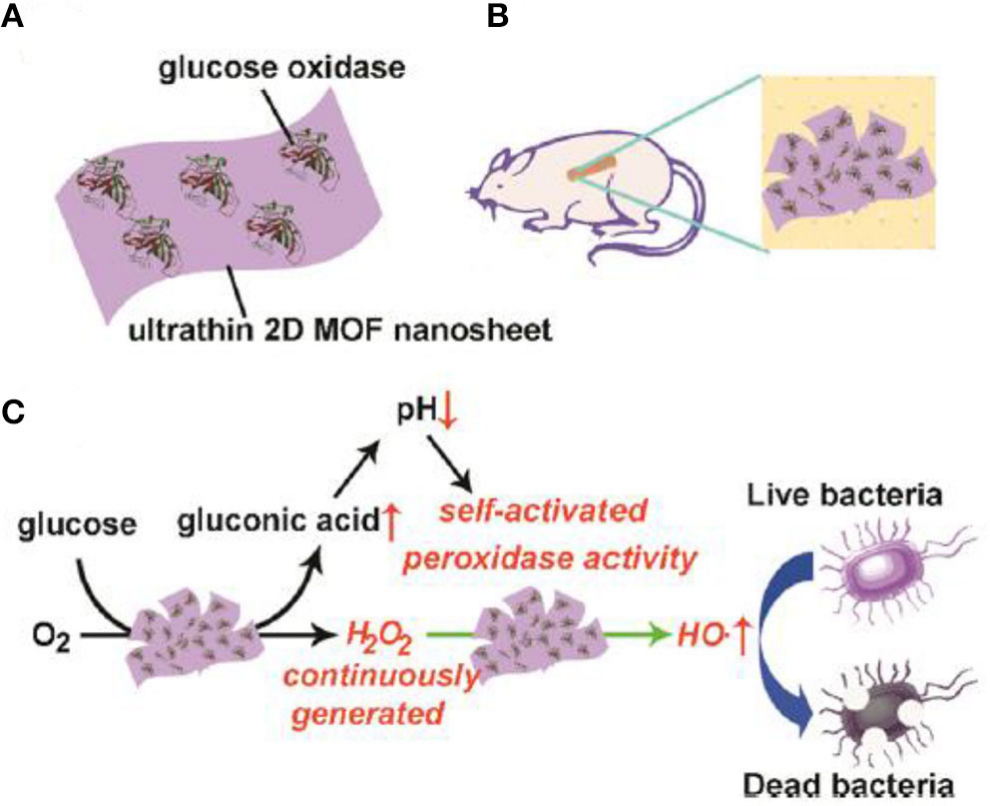
Illustration of **(A)** composition, **(B)** use for wound healing of mice, and **(C)** antibacterial mechanism of 2D MOF/GOx hybrid nanozyme. Reproduced with permission from Liu X. P. et al. (2019). Copyright 2019 American Chemical Society.

Recently, Qu's group constructed a self-activated cascade reagent based on GOx-adsorbed 2D Cu-TCPP(Fe) MOF NSs (Figure 2A), which was used together with glucose in the antibacterial system (Liu X. P. et al., 2019). The authors also created the wound model on the back of Kunming mice and prepared a MOF/GOx-band-aid for the *in vivo* bacterial study (Figure 2B). The principle was based on the consecutive reactions, in which GOx firstly catalyzed the oxidation of glucose to gluconic acid and H_2_O_2_, which was further catalytically decomposed into ·OH radicals by MOF NSs as a POD-mimic, thus leading to an antibacterial effect and ultimately, wound healing (Figure 2C). In the *in vitro* antibacterial investigation, the group (glucose + MOF/GOx) led to high bacteria inactivation rates, up to 88% and 90 % for *E. Coli* and *S. aureus*, respectively. By contrast, for the control groups including (1) PBS, (2) glucose, (3) glucose + MOF, and (4) MOF/GOx, the viabilities remained above 50% for the two bacteria. In the *in vivo* antibacterial study, the bacteria for the group (glucose + MOF/GOx-band-aid) were decreased to 9.1%; however, the bacteria for the control group (glucose + GOx-band-aid) were only decreased to 56.5%. These results showed high antibacterial activity of MOF/GOx. More interestingly, since H_2_O_2_ is an oxidant that was continuously produced by oxidation of glucose in the cascade catalysis, the study provided a benign antibacterial system to avoid the direct introduction of highly concentrated H_2_O_2_.

#### Antioxidants

2D NMs had important applications in antioxidants. Table 3 shows a summary of 2D nanozymes as antioxidants. Typical examples include GO NSs (Halim et al., 2019), Se NPs *in situ* grown on GO NSs (Huang et al., 2017b), and MoS_2_ NSs prepared by sonication-assisted exfoliation (Chen T. M. et al., 2018). In another example, the different TMDs (WS_2_, MoSe_2_, and WSe_2_) were functionalized with an amphiphilic poly(ε-caprolactone)-*b*-PEG (PCL-*b*-PEG) diblock copolymer, which efficiently captured mitochondrial and intracellular ROS and reaction nitrogen species (RNS) like ·NO radicals, of which the WS_2_ NSs displayed the best performance (Yim et al., 2020). With effective scavenging of ROS and RNS as well as suppression of inflammatory cytokines of WS_2_ NSs, the survival rate of the septic mice remarkably increased to 90%.

**Table 3:** Summary of representative 2D nanozymes as antioxidants.

**2D NMs**	**Enzymatic activity**	**Applications**	**References**
GO NSs	SOD, CAT	Protecting mesenchymal stem cells from ROS accumulation	Halim et al., 2019
Se/GO	Glutathione Peroxidase	protecting RAW264.7 cells from oxidative stress by catalyzing H_2_O_2_ decomposition to H_2_O	Huang et al., 2017
MoS_2_ NSs	SOD, CAT	Protecting *Escherichia coli* (*E. coli*), *Staphylococcus aureus* (*S. aureus*) and A549 cells from H_2_O_2_-induced oxidative stress	Chen T. M. et al., 2018
WS_2_, MoSe_2_ and WSe_2_ NSs coated with PCL-b-PEG	SOD, CAT	Scavenging mitochondrial and intracellular ROS and RNS in lipopolysaccharide (LPS)- or bacteria-induced inflammatory cells	Yim et al., 2020
PVP-modified Nb_2_C NSs	SOD	Scavenging ROS against ionizing radiation	Ren et al., 2019
Se-modified *g*-C_3_N_4_ NSs	CAT	Protecting A549 cells from ROS-induced damage	Cao X. N. et al., 2020

Chen's group found that the PVP-functionalized Nb_2_C NSs could greatly reduce ROS generation caused by ionizing radiation, thereby providing a kind of antioxidant based on 2D nanozymes for radioprotective applications (Ren et al., 2019). Wang's group also demonstrated the potential of Se-modified *g*-C_3_N_4_ NSs against oxidative stress (Cao X. N. et al., 2020).

#### Therapeutics

**Figure 3:**
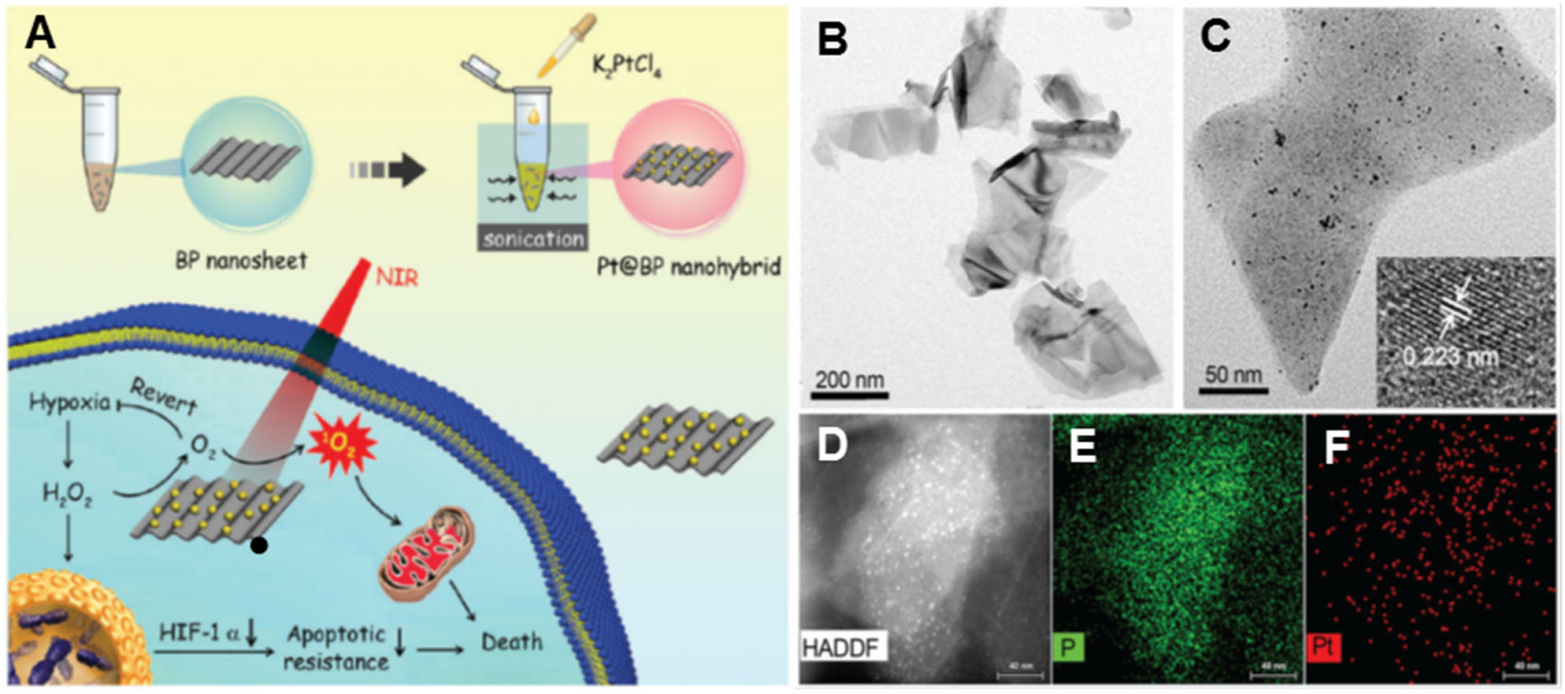
**(A)** Illustration of synthesis and antitumor process of Pt/BP nanocomposites as well as their characterization: **(B)** TEM image of BP NSs and **(C)** Pt/BP nanocomposites (inset shows the high-resolution TEM image of a Pt NP), **(D)** high-angle annular dark-field scanning TEM (HAADF-STEM) image of Pt/BP, elemental maps of **(E)** P, and **(F)** Pt. Adapted with permission from Ouyang et al. (2018). Copyright 2018 Royal Society of Chemistry.

A new hotspot in nanozyme research is the integration of enzymatic properties for therapy. Using an *in situ* growth strategy, Liu's group fabricated Pt NPs decorated on the surface of black phosphorus (BP) NSs, obtained by the liquid exfoliation method (Figure 3A) (Ouyang et al., 2018). The as-obtained BP NSs were freestanding with several hundred nanometers (Figure 3B), while the Pt NPs had an average size of appropriately 4.2 nm (Figure 3C), with the crystal lattice fringe of 0.223 nm of (111) plane. The element distribution was further obtained from high-angle annular dark-field scanning TEM (HAADF-STEM) image (Figure 3D) and element maps (Figures 3E,F). The as-obtained Pt/BP nanocomposites integrated CAT-like activity of Pt NPs and photodynamic therapy (PDT) activity of BP NSs, in which the Pt NPs catalytically decomposed the accumulated H_2_O_2_ in tumors to relieve tumor hypoxia (Figure 3A). In the *in vitro* antitumor experiment, the BP NSs alone only reduced 26% of cell viability under near infrared (NIR) light irradiation, while about 65% of tumor cells were dead after the treatment of the Pt/BP. The result showed that the elevated O_2_ level caused by CAT-catalysis of Pt NPs remarkably improved the PDT efficiency of BP NSs. Moreover, as a result of the Pt/BP treatment, the overexpression of hypoxia-inducible factor-1α (HIF-1α), which is associated with therapy resistance in tumor cells, was significantly down-regulated, since the intensity of fluorescence appeared on the tumor slices of mice was decreased by 64% in the immunohistochemical staining experiment. The down-regulation of HIF-1α by the Pt/BP decreased the tumor apoptotic resistance, which ultimately enhanced the therapeutic effect in the *in vivo* antitumor experiment, in which the growth of the tumor was completely regressed by the treatment of Pt/BP, while the treatment of BP NSs alone only led to a decline in about 47% of average tumor size.

By using a similar *in situ* growth method, Sun's group synthesized Pt NPs coated 2D MOF, that is, Sm-modified tetrakis(4-carboxyphenyl)porphyrin (Sm-TCPP), which afforded a multifunctional nanozyme with excellent tumor specificity for enhanced PDT efficiency (Cao Z. G. et al., 2020). In their study, the Pt NPs acted as a CAT-mimic, catalyzing over-expressed H_2_O_2_ in a tumor microenvironment (TME) into O_2_. The generated O_2_ was further catalytically transformed to ^1^O_2_ by Sm-TCPP, owing to its high ROS generation capacity under light irradiation.

## Prospect and Challenges

This review has undertaken a summary of recent progress in developing 2D NMs with enzyme-like activities for diversified biomedical applications. The continuous advancement of nanoscience and nanotechnology in relation to 2D NMs will offer great opportunities to develop new types of nanozymes with varied functionalities in the future. Meanwhile, the exploration of new biocatalytic properties and integration of enzymatic activities of 2D NMs with other nanostructures like 0D NPs have potentially limitless applications in biomedical areas, and more fundamental technological breakthroughs in 2D nanozymes are expected in the near future.

Despite the many achievements that have been made in research on 2D nanozymes, studies on this field are still in the initial stages and future studies must address some challenges. Firstly, compared to 0D NPs with easy preparation, it is challenging to achieve the controlled synthesis of 2D NMs with uniform thickness, desirable size, and colloidal stability. Despite great success in creating layered materials by the top-down methods, there are still some obvious shortcomings. A typical example is the synthesis of TMDs for biomedical applications, in which the liquid exfoliation and chemical intercalation methods were adopted in most studies. Although the former had a high yield with simple operations, the morphologies of the obtained products had a lack of uniformity, often involving a mixture of single and few-layer sheets with different lateral dimensions (Agarwal and Chatterjee, 2018). Despite the more uniform single-layer NSs created by the latter, it both brought structural and electronic deformations and involved toxic reagents. The chemical intercalation process was highly sensitive to the environment and time-consuming. Therefore, it is still desirable to develop an efficient method to prepare TMDs. Another example is the metallic NSs. Bearing lots of unsaturated atoms, the ultrathin metallic nanostructures are difficult to stabilize, because metal atoms readily form 3D close-packed crystals. Despite tremendous efforts in the synthesis of metallic NSs by bottom-up protocols, the formation mechanism in most reports was proposed based on a simple correlation between a few synthetic parameters and the final shape, in which several characterization techniques (e.g., TEM, XRD, etc.) were employed to identify reaction intermediates, were formed either at certain time intervals or in the control experiments (Chen Y. et al., 2018). However, there is not enough evidence relating to authentic reactive species during synthesis. Therefore, to better understand the formation process (e.g., nucleation, growth, and assembly) of metallic NSs for synthesis, it is essential to carry out more *in situ* studies on the morphological evolution toward the 2D structure.

Secondly, despite the fascinating enzymatic properties of numerous 2D NMs, few studies on the in-depth catalytic mechanism are available. At present, it is still difficult to predict the nanozyme activity of 2D NMs for a specific structure. It is also difficult to achieve the best performance (e.g., activity and selectivity) for a specific application in biomedical areas, and hard to design the desired 2D nanozymes with optimal structures. Fundamentally, the missing link of structure-property relationship is largely because the enzyme-catalysis mechanism of 2D NMs remains unclear. For example, we must ask what the true active sites of 2D NMs and catalytically active intermediates during enzyme-catalysis are. In this regard, a deeper understanding of the catalytic mechanism of 2D nanozymes could benefit from *in situ* experimental studies and theoretical calculation by establishing appropriate models for catalysis, which are beneficial to the structural design of 2D nanozymes.

Thirdly, unlike 0D nanosystems, the biocompatibility and environmental stability of 2D NMs remains largely unknown, to date. Despite numerous studies on the biocompatibility of graphene-based NMs and TMDSs, evaluations of the toxicity of other types of 2D NMs are urgently required. The solubility, biodegradation, and biocompatibility of 2D NMs could be different from each other due to differences in their structure, composition, the methodology of synthesis and functionalization, and the cells studied. Therefore, it is hard to speculate whether a specific 2D NM is toxic or not. Their toxicity (both short-term and long-term), cellular-uptake mechanism, and the metabolic pathways of 2D NMs should be systematically tested for clinical trials.

Finally, it is acknowledged that many of the current applications of 2D nanozymes that display an outstanding performance are just proof-of-concept and have only been performed in laboratories. A number of reports fail to examine processability, scale-up possibility, and cost. The long-term stability and durability of 2D nanozymes are also less explored by researchers. Accordingly, there is still a long way to go before 2D nanozymes can be implemented in practical applications, which ultimately require interdisciplinary collaboration from chemistry, materials science, and biology.

## Author Contributions

RY: design the work. SC: drafting the manuscript. All authors contributed to the article and approved the submitted version.

## Conflict of Interest

The authors declare that the research was conducted in the absence of any commercial or financial relationships that could be construed as a potential conflict of interest. The handling Editor declared a shared affiliation, though no other collaboration, with one of the authors SC and RY.

## References

[B1] Agarwal V. Chatterjee K. (2018). Recent advances in the field of transition metal dichalcogenides for biomedical applications. Nanoscale 10, 16365–16397. 10.1039/C8NR04284E 30151537

[B2] Allen M. J. Tung V. C. Kaner R. B. (2010). Honeycomb carbon: a review of graphene. Chem. Rev. 110, 132–145. 10.1021/cr900070d 19610631

[B3] Bhimanapati G. R. Lin Z. Meunier V. Jung Y. Cha J. Das S.. (2015). Recent advances in two-dimensional materials beyond graphene. ACS Nano. 9, 11509–11539. 10.1021/acsnano.5b05556 26544756

[B4] Butler S. Z. Hollen S. M. Cao L. Y. Cui Y. Gupta J. A. Gutiérrez H. R.. (2013). Progress, challenges, and opportunities in two-dimensional materials beyond graphene. ACS Nano. 7, 2898–2926. 10.1021/nn400280c 23464873

[B5] Cai S. F. Fu Z. Xiao W. Xiong Y. L. Wang C. Yang R. (2020). Zero-dimensional/two-dimensional Au_*x*_Pd_100−x_ nanocomposites with enhanced nanozyme catalysis for sensitive glucose detection. ACS Appl. Mater. Interfaces. 12, 11616–11624. 10.1021/acsami.9b21621 32068379

[B6] Cai S. F. Han Q. S. Qi C. Lian Z. Jia X. H. Yang R.. (2016). Pt_74_Ag_26_ nanoparticle-decorated ultrathin mos_2_ nanosheets as novel peroxidase mimics for highly selective colorimetric detection of H_2_O_2_ and glucose. Nanoscale 8, 3685–3693. 10.1039/C5NR08038J 26811962

[B7] Cai S. F. Han Q. S. Qi C. Wang X. H. Wang T. Jia X. H. (2017). MoS_2_-Pt_3_Au_1_ nanocomposites with enhanced peroxidase-like activities for selective colorimetric detection of phenol. Chin. J. Chem. 35, 605–612. 10.1002/cjoc.201600694

[B8] Cai S. F. Lian C. Duan H. H. Xiao W. Han Q. S. Qi C. (2019). Facile strategy to prepare Rh nanosheet-supported PtRh nanoparticles with synergistically enhanced catalysis in oxidation. Chem. Mater. 31, 808–818. 10.1021/acs.chemmater.8b03889

[B9] Cai S. F. Liu X. L. Han Q. S. Qi C. Yang R. Wang C. (2018a). A novel strategy to construct supported Pd nanocomposites with synergistically enhanced catalytic performances. Nano Res. 11, 3272–3281. 10.1007/s12274-017-1868-9

[B10] Cai S. F. Xiao W. Duan H. H. Liang X. X. Wang C. Yang R. (2018b). Single-layer Rh nanosheets with ultrahigh peroxidase-like activity for colorimetric biosensing. Nano Res. 11, 6304–6315. 10.1007/s12274-018-2154-1

[B11] Cai S. F. Yang R. (2020). Noble Metal-Based Nanozymes. in: Nanozymology. Nanostructure Science and Technology, eds X. Y. Yan (Singapore: Springer), 331–365. 10.1007/978-981-15-1490-6_10

[B12] Cao X. N. Lian S. Tong Y. W. Lin W. Jia L. Fang Y. X.. (2020). Fluorescent Se-modified carbon nitride nanosheets as biomimetic catalases for free-radical scavenging. Chem. Commun. 56, 916–919. 10.1039/C9CC08665J 31850457

[B13] Cao Z. G. Li Y. J. Zhang Y. Cheng K. W. An P. J. Chen F. H. (2020). Biomimetic platinum nanozyme immobilized on 2D metal-organic frameworks for mitochondrion-targeting and oxygen self-supply photodynamic therapy. ACS Appl. Mater. Interfaces 12, 1963–1972. 10.1021/acsami.9b14958 31873002

[B14] Chen J. Y. Shu Y. Li H. L. Xu Q. Hu X. Y. (2018). Nickel metal-organic framework 2D nanosheets with enhanced peroxidase nanozyme activity for colorimetric detection of H_2_O_2_. Talanta 189, 254–261. 10.1016/j.talanta.2018.06.075 30086915

[B15] Chen M. M. Wei D. Chu W. Wang T. Tong D. G. (2017). One-pot synthesis of O-doped BN nanosheets as a capacitive deionization electrode for efficient removal of heavy metal ions from water. J. Mater. Chem. A 5, 17029–17039. 10.1039/C7TA05459A

[B16] Chen T. M. Wu X. J. Wang J. X. Yang G. W. (2017). WSe_2_ few layers with enzyme mimic activity of high-sensitive and high-selective visual detection of glucose. Nanoscale 9, 11806–11813. 10.1039/C7NR03179C 28786467

[B17] Chen T. M. Zou H. Wu X. J. Liu C. C. Situ B. Zheng L.. (2018). Nanozymatic antioxidant system based on MoS_2_ nanosheets. ACS Appl. Mater. Interfaces. 10, 12453–12462. 10.1021/acsami.8b01245 29595050

[B18] Chen Y. Fan Z. X. Zhang Z. C. Niu W. X. Li C. L. Yang N. L.. (2018). Two-dimensional metal nanomaterials: synthesis, properties, and applications. Chem. Rev. 118, 6409–6455. 10.1021/acs.chemrev.7b00727 29927583

[B19] Chen Y. Tan C. L. Zhang H. Wang L. Z. (2015). Two-dimensional graphene analogues for biomedical applications. Chem. Soc. Rev. 44, 2681–2701. 10.1039/C4CS00300D 25519856

[B20] Cheng L. Liu J. J. Gu X. Gong H. Shi X. Z. Liu T.. (2014). PEGylated WS_2_ nanosheets as a multifunctional theranostic agent for *in vivo* dual-modal CT/photoacoustic imaging guided photothermal therapy. Adv. Mater. 26, 1886–1893. 10.1002/adma.201304497 24375758

[B21] Chimene D. Alge D. L. Gaharwar A. K. (2015). Two-dimensional nanomaterials for biomedical applications: emerging trends and future prospects. Adv. Mater. 27, 7261–7284. 10.1002/adma.201502422 26459239

[B22] Choleva T. G. Gatselou V. A. Tsogas G. Z. Giokas D. L. (2018). Intrinsic peroxidase-like activity of rhodium nanoparticles, and their application to the colorimetric determination of hydrogen peroxide and glucose. Microchim. Acta. 185, 22–30. 10.1007/s00604-017-2582-8 29594622

[B23] Coleman J. N. Lotya M. O'Neill A. Bergin S. D. King P. J. Khan U.. (2011). Two-dimensional nanosheets produced by liquid exfoliation of layered materials. Science 331, 568–571. 10.1126/science.1194975 21292974

[B24] Darabdhara G. Bordoloi J. Manna P. Das M. R. (2019). Biocompatible bimetallic Au-Ni doped graphitic carbon nitride sheets: a novel peroxidase-mimicking artificial enzyme for rapid and highly sensitive colorimetric detection of glucose. Sens. Actuat. B 285, 277–290. 10.1016/j.snb.2019.01.048

[B25] Ding Y. J. Chen Y. P. Zhang X. L. Chen L. Dong Z. H. Jiang H. L.. (2017). Controlled intercalation and chemical exfoliation of layered metal-organic frameworks using a chemically labile intercalating agent. J. Am. Chem. Soc. 139, 9136–9139. 10.1021/jacs.7b04829 28651432

[B26] Dong Y. Q. Wang Q. Wu H. S. Chen Y. M. Lu C.-H. Chi Y. W.. (2018). Graphitic carbon nitride materials: sensing, imaging and therapy. Biomater. Sci. 6, 2298–2311. 10.1002/smll.201602056 27611869

[B27] Duan H. H. Yan N. Yu R. Chang C. R. Zhou G. Hu H. S.. (2014). Ultrathin rhodium nanosheets. Nat. Commun. 5:3093. 10.1038/ncomms4093 24435210

[B28] Dutta S. Ray C. Mallick S. Sarkar S. Sahoo R. Negishi Y. (2015). A gel-based approach to design hierarchical CuS decorated reduced graphene oxide nanosheets for enhanced peroxidase-like activity leading to colorimetric detection of dopamine. J. Phys. Chem. C. 119, 23790–23800. 10.1021/acs.jpcc.5b08421

[B29] Feng L. P. Zhang L. X. Zhang S. Chen X. Li P. Gao Y.. (2020). Plasma-assisted controllable doping of nitrogen into MoS_2_ nanosheets as efficient nanozymes with enhanced peroxidase-like catalysis activity. ACS Appl. Mater. Interfaces 12, 17547–17556. 10.1021/acsami.0c01789 32223269

[B30] Gan X. R. Zhao H. M. Quan X. (2017). Two-dimensional MoS_2_: a promising building block for biosensors. Biosens. Bioelectron. 89, 56–71. 10.1016/j.bios.2016.03.042 27037158

[B31] Gao F. Yang X. Luo X. P. Xue X. L. Qian C. G. Sun M. J. (2020). Photoactivated nanosheets accelerate nucleus access of cisplatin for drug-resistant cancer therapy. Adv. Funct. Mater. 2001546. 10.1002/adfm.202001546

[B32] Gao L. Z. Zhuang J. Nie L. Zhang J. B. Zhang Y. Gu N.. (2007). Intrinsic peroxidase-like activity of ferromagnetic nanoparticles. Nat. Nanotechnol. 2, 577–583. 10.1038/nnano.2007.260 18654371

[B33] Ge J. Cai R. Chen X. G. Wu Q. Zhang L. L. Jiang Y.. (2019). Facile approach to prepare HSA-templated MnO_2_ nanosheets as oxidase mimic for colorimetric detection of glutathione. Talanta 195, 40–45. 10.1016/j.talanta.2018.11.024 30625560

[B34] Ghosal K. Sarkar K. (2018). Biomedical applications of graphene nanomaterials and beyond. ACS Biomater. Sci. Eng. 4, 2653–2703. 10.1021/acsbiomaterials.8b00376 33434995

[B35] Girish C. M. Sasidharan A. Gowd G. S. Nair S. Koyakutty M. (2013). Confocal Raman imaging study showing macrophage mediated biodegradation of graphene *in vivo*. Adv. Healthcare Mater. 2, 1489–1500. 10.1002/adhm.201200489 23554400

[B36] Gollavelli G. Ling Y.-C. (2012). Multi-functional graphene as an *in vitro* and *in vivo* imaging probe. Biomaterials 33, 2532–2545. 10.1016/j.biomaterials.2011.12.010 22206596

[B37] Guan G. J. Han M.-Y. (2019). Functionalized hybridization of 2D nanomaterials. Adv. Sci. 6:1901837 10.1002/advs.201901837 PMC6891915 31832321

[B38] Halim A. Liu L. Ariyanti A. D. Ju Y. Luo Q. Song G. B. (2019). Low-dose suspended graphene oxide nanosheets induce antioxidant response and osteogenic differentiation of bone marrow-derived mesenchymal stem cells via JNK-dependent FoxO1 activation. J. Mater. Chem. B. 7, 5998–6009. 10.1039/C9TB01413F 31538158

[B39] Harvey A. He X. Y. Godwin I. J. Backes C. McAteer D. Berner N. C. (2016). Production of Ni(OH)_2_ nanosheets by liquid phase exfoliation: from optical properties to electrochemical applications. J. Mater. Chem. A 4, 11046–11059. 10.1039/C6TA02811J

[B40] Hasan M. A. Zaki M. I. Pasupulety L. Kumari K. (1999). Promotion of the hydrogen peroxide decomposition activity of manganese oxide catalysts. Appl. Catal. A 181:171 10.1016/S0926-860X(98)00430-X

[B41] He W. Jia H. Li X. Lei Y. Li J. Zhao H.. (2012). Understanding the formation of CuS concave superstructures with peroxidase-like activity. Nanoscale 4, 3501–3506. 10.1039/c2nr30310h 22552534

[B42] He W. W. Wu X. C. Liu J. B. Hu X. N. Zhang K. Hou S. (2010). Design of AgM bimetallic alloy nanostructures (M = Au, Pd, Pt) with tunable morphology and peroxidase-like activity. Chem. Mater. 22, 2988–2994. 10.1021/cm100393v

[B43] Heo N. S. Song H. P. Lee S. M. Cho H. J. Kim H. J. Huh Y. S.. (2020). Rosette-shaped graphitic carbon nitride acts as a peroxidase mimic in a wide pH range for fluorescence-based determination of glucose with glucose oxidase. Microchim. Acta. 187:286. 10.1007/s00604-020-04249-z 32328802

[B44] Hu T. T. Mei X. Wang Y. J. Weng X. S. Liang R. Z. Wei M. (2019). Two-dimensional nanomaterials: fascinating materials in biomedical field. Sci. Bull. 64, 1707–1727. 10.1016/j.scib.2019.09.021 36659785

[B45] Huang J. K. Zhang J. Z. Shi G.-W. Wei; Liu Y. M. (2014). Hydrothermal synthesis of molybdenum disulfide nanosheets as supercapacitors electrode material. Electrochim. Acta. 132, 397–403. 10.1016/j.electacta.2014.04.007

[B46] Huang K. Li Z. J. Lin J. Han G. Huang P. (2016). Two-dimensional transition metal carbides and nitrides (MXenes) for biomedical applications. Chem. Soc. Rev. 47, 5109–5124. Small 12, 5376–5393. 10.1039/C7CS00838D 29667670

[B47] Huang L. J. Zhu W. X. Zhang W. T. Chen K. Wang J. Wang R.. (2018). Layered Vanadium(IV) disulfide nanosheets as a peroxidase-like nanozyme for colorimetric detection of glucose. Microchim. Acta. 185, 7–15. 10.1007/s00604-017-2552-1 29594417

[B48] Huang X.-W. Wei J. J. Liu T. Zhang X. L. Bai S. M. Yang H. H. (2017). Silk fibroin-assisted exfoliation and functionalization of transition metal dichalcogenide nanosheets for antibacterial wound dressings. Nanoscale 9, 17193–17198. 10.1039/C7NR06807G 29095468

[B49] Huang X. Q. Tang S. H. Mu X. L. Dai Y. Chen G. X. Zhou Z. Y.. (2011). Freestanding palladium nanosheets with plasmonic and catalytic properties. Nat. Nanotechnol. 6, 28–32. 10.1038/nnano.2010.235 21131956

[B50] Huang Y. Liu C. Q. Pu F. Liu Z. Ren J. S. Qu X. G. (2017b). A Go-Se nanocomposites as an antioxidant nanozyme for cytoprotection. Chem. Commun. 53, 3082–3085. 10.1039/C7CC00045F 28243649

[B51] Huang Y. Zhao M. T. Han S. K. Lai Z. C. Yang J. Tan C. L.. (2017a). Growth of Au nanoparticles on 2D metalloporphyrinic metal-organic framework nanosheets used as biomimetic catalysts for cascade reactions. Adv. Mater. 29:1700102. 10.1002/adma.201700102 28634989

[B52] Huang Y. Y. Ren J. S. Qu X. G. (2019). Nanozymes: classification, catalytic mechanisms, activity regulation, and applications. Chem. Rev. 119, 4357–4412. 10.1021/acs.chemrev.8b00672 30801188

[B53] Huo C. Yan Z. Song X. (2015). 2D materials via liquid exfoliation: a review on fabrication and applications. Sci. Bull. 60, 1994–2008. 10.1007/s11434-015-0936-3

[B54] Ivanova M. N. Grayfer E. D. Plotnikova E. E. Kibis L. S. Darabdhara G. Boruah P. K.. (2019). Pt-decorated boron nitride nanosheets as artificial nanozyme for detection of dopamine. ACS Appl. Mater. Interfaces 11, 22102–22112. 10.1021/acsami.9b04144 31124654

[B55] Jiang C. M. Wu C. Li X. J. Yao Y. Lan L. Y. Zhao F. N. (2019). All-electrospun flexible triboelectric nanogenerator based on metallic MXene nanosheet. Nano Energy. 59, 268–276. 10.1016/j.nanoen.2019.02.052

[B56] Jiang D. W. Ni D. L. Rosenkrans Z. T. Huang P. Yan X. Y. Cai W. B. (2019). Nanozyme: new horizons for responsive biomedical applications. Chem. Soc. Rev. 48, 3683–3704. 10.1039/C8CS00718G 31119258 PMC6696937

[B57] Jiao L. Yan H. Y. Wu Y. Gu W. L. Zhu C. Z. Du D.. (2019). When nanozymes meet single-atom catalysis. Angew. Chem. Int. Ed. 59, 2565–2576. 10.1002/anie.201905645 31209985

[B58] Ju Y. Kim J. (2015). Dendrimer-encapsulated Pt nanoparticles with peroxidase-mimetic activity as biocatalytic labels for sensitive colorimetric analyses. Chem. Commun. 51, 13752–13755. 10.1039/C5CC06055A 26238303

[B59] Khan K. Tareen A. K. Aslam M. Wang R. H. Zhang Y. P. Mahmood A. (2020). Recent developments in emerging two-dimensional materials and their applications. J. Mater. Chem. C. 8, 387–440. 10.1039/C9TC04187G

[B60] Kim M. S. Cho S. Joo S. H. Lee J. Kwak S. K. Kim M. I.. (2019). N- and B-codoped graphene: a strong candidate to replace natural peroxidase in sensitive and selective bioassays. ACS Nano. 13, 4312–4321. 10.1021/acsnano.8b09519 30908007

[B61] Kim M. S. Lee J. Kim H. S. Cho A. Shim K. H. Le T. N. (2020). Heme cofactor-resembling Fe-N single site embedded graphene as nanozymes to selectively detect H_2_O_2_ with high sensitivity. Adv. Funct. Mater. 30:1905410 10.1002/adfm.201905410

[B62] Kong L. Xing L. Zhou B. Du L. Shi X. (2017). Dendrimer-modified MoS_2_ nanoflakes as a platform for combinational gene silencing and photothermal therapy of tumors. ACS Appl. Mater. Interfaces 9, 15995–16005. 10.1021/acsami.7b03371 28441474

[B63] Korschelt K. Ragg R. Metzger C. S. Kluenker M. Oster M. Barton B.. (2017). Glycine-functionalized copper(II) hydroxide nanoparticles with high intrinsic superoxide dismutase activity. Nanoscale 9, 3952–3960. 10.1039/C6NR09810J 28265620

[B64] Korsvik C. Patil S. Seal S. Self W. T. (2007). Superoxide dismutase mimetic properties exhibited by vacancy engineered ceria nanoparticles. Chem. Commun. 1056–1058. 10.1039/b615134e 17325804

[B65] Li D. Muller M. B. Gilje S. Kaner R. B. Wallace G. G. (2008). Processable aqueous dispersions of graphene nanosheets. Nat. Nanotechnol. 3, 101–105. 10.1038/nnano.2007.451 18654470

[B66] Li Z. H. Liu X. Y. Liang X. H. Zhong J. H. Guo L. Q. Fu F. F. (2019). Colorimetric determination of xanthine in urine based on peroxidase-like activity of WO_3_ nanosheets. Talanta 204, 278–284. 10.1016/j.talanta.2019.06.003 31357294

[B67] Liang Q. H. Li Z. Bai Y. Huang Z.-H. Kang F. Y. Yang Q.-H. (2017). Reduced-sized monolayer carbon nitride nanosheets for highly improved photoresponse for cell imaging and photocatalysis. Sci. China Mater. 60, 109–118. 10.1007/s40843-016-5131-9

[B68] Liao K.-H. Lin Y.-S. Macosko C. W. Haynes C. L. (2011). Cytotoxicity of graphene oxide and graphene in human erythrocytes and Skin Fibroblasts. ACS Appl. Mater. Interfaces 3, 2607–2615. 10.1021/am200428v 21650218

[B69] Lin H. Wang X. G. Yu L. D. Chen Y. Shi J. L. (2017). Two-dimensional ultrathin MXene ceramic nanosheets for photothermal conversion. Nano Lett. 17, 384–391. 10.1021/acs.nanolett.6b04339 28026960

[B70] Lin S. S. Gurol M. D. (1998). Catalytic decomposition of hydrogen peroxide on iron oxide: kinetics, mechanism, and implication. Environ. Sci. Technol. 32, 1417–1423. 10.1021/es970648k

[B71] Lin T. R. Zhong L. S. Guo L. Q. Fu F. F. Chen G. N. (2014a). Seeing diabetes: visual detection of glucose based on the intrinsic peroxidase-like activity of MoS_2_ nanosheets. Nanoscale 6, 11856–11862. 10.1039/C4NR03393K 25171261

[B72] Lin T. R. Zhong L. S. Song Z. P. Guo L. Q. Wu H. Y. Guo Q. Q.. (2014b). Visual detection of blood glucose based on peroxidase-like activity of WS_2_ nanosheets. Biosens. Bioelectron. 62, 302–307. 10.1016/j.bios.2014.07.001 25032681

[B73] Lin T. R. Zhong L. S. Wang J. Guo L. Q. Wu H. Y. Guo Q. Q.. (2014c). Graphite-like carbon nitrides as peroxidase mimetics and their applications to glucose detection. Biosens. Bioelectron. 59, 89–93. 10.1016/j.bios.2014.03.023 24704762

[B74] Liu J. Meng L. J. Fei Z. F. Dyson P. J. Jing X. N. Liu X. (2017). MnO_2_ nanosheets as an artificial enzyme to mimic oxidase for rapid and sensitive detection of glutathione. Biosens. Bioelectron. 90, 69–74. 10.1016/j.bios.2016.11.046 27886603

[B75] Liu J.-H. Yang S.-T. Wang H. Chang Y. Cao A. Liu Y. (2012). Effect of size and dose on the biodistribution of graphene oxide in mice. Nanomedicine 7, 1801–1812. 10.2217/nnm.12.60 22830500

[B76] Liu T. Wang C. Gu X. Gong H. Cheng L. Shi X. Z.. (2014). Drug delivery with PEGylated MoS_2_ nano-sheets for combined photothermal and chemotherapy of cancer. Adv. Mater. 26, 3433–3440. 10.1002/adma.201305256 24677423

[B77] Liu X. L. Wang X. H. Han Q. S. Qi C. Wang C. Yang R. (2019). Facile synthesis of IrO_2_/rGO nanocomposites with high peroxidase-like activity for sensitive colorimetric detection of low weight biothiols. Talanta 203, 227–234. 10.1016/j.talanta.2019.05.070 31202330

[B78] Liu X. N. Huang L. J. Wang Y. P. Sun J. Yue T. L. Zhang W. T. (2020). One-pot bottom-up fabrication of a 2D/2D heterojuncted nanozyme towards optimized peroxidase-like activity for sulfide ions sensing. Sens. Actuat. B 306:127565 10.1016/j.snb.2019.127565

[B79] Liu X. P. Yan Z. Q. Zhang Y. Liu Z. W. Sun Y. H. Ren J. S.. (2019). Two-dimensional metal-organic framework/enzyme hybrid nanocatalyst as a benign and self-activated cascade reagent for *in vivo* wound healing. ACS Nano. 13, 5222–5230. 10.1021/acsnano.8b09501 31002497

[B80] Liu Y. Wu H. H. Li M. Yin J.-J. Nie Z. H. (2014). pH dependent catalytic activities of platinum nanoparticles with respect to the decomposition of hydrogen peroxide and scavenging of superoxide and singlet oxygen. Nanoscale 6, 11904–11910. 10.1039/C4NR03848G 25175625

[B81] Lu X. L. Feng X. D. Werber J. R. Chu C. H. Zucker I. Kim J. H.. (2017). Enhanced antibacterial activity through the controlled alignment of graphene oxide nanosheets. Proc. Natl. Acad. Sci. U. S. A. 114, E9793–E9801. 10.1073/pnas.1710996114 29078354 PMC5699062

[B82] Ma D. Q. Xie C. J. Wang T. Mei L. Q. Zhang X. Guo Z.. (2020). Liquid-phase exfoliation and functionalization of MoS_2_ nanosheets for effective antibacterial application. ChemBioChem. 21, 2373–2380. 10.1002/cbic.202000195 32227558

[B83] Ma D. T. Zhao J. L. Xie J. L. Zhang F. Wang R. Wu L. M.. (2020). Ultrathin boron nanosheets as an emerging two-dimensional photoluminescence material for bioimaging. Nanoscale Horiz. 5, 705–713. 10.1039/C9NH00698B 32226968

[B84] Maromeze C. M. Dos Santos G. P. De Moraes V. B. Da C. L. Kubota L. T. (2016). Multifunctional catalytic platform for peroxidase mimicking, enzyme immobilization and biosensing. Biosens. Bioelectron. 77, 746–751. 10.1016/j.bios.2015.10.042 26499871

[B85] Mateti S. Wong C. S. Liu Z. Yang W. R. Li Y. C. Li L. H. (2018). Biocompatibility of boron nitride nanosheets. Nano Res. 11, 334–342. 10.1007/s12274-017-1635-y

[B86] Merlo A. Mokkapati V. R. S. S. Pandit S. Mijakovic I. (2018). Boron nitride nanomaterials: biocompatibility and bio-applications. Biomater. Sci. 6, 2298–2311. 10.1039/C8BM00516H 30059084

[B87] Mu J. S. Wang Y. Zhao M. Zhang L. (2012). Intrinsic peroxidase-like activity and catalase-like activity of Co_3_O_4_ nanoparticles. Chem. Commun. 48, 2540–2542. 10.1039/c2cc17013b 22288077

[B88] Mu J. S. Zhang L. Zhao M. Wang Y. (2013). Co_3_O_4_ nanoparticles as an efficient catalase mimic: properties, mechanism and its electrocatalytic sensing application for hydrogen peroxidase. J. Mol. Catal. A Chem. 378, 30–37. 10.1016/j.molcata.2013.05.016

[B89] Novoselov K. S. Geim A. K. Morozov S. V. Jiang D. Zhang Y. Dubonos S. V. (2004). Electric field effect in atomically thin carbon films. Science 306, 666–669. 10.1126/science.1102896 15499015

[B90] Novoselov K. S. Jiang D. Schedin F. Booth T. J. Khotkevich V. V. Morozov S. V.. (2005). Two-dimensional atomic crystals. *Proc. Natl. Acad. Sci*. U. S. A. 102, 10451–10453. 10.1073/pnas.0502848102 16027370 PMC1180777

[B91] Oudeng G. Au M. Shi J. Wen C. Yang M. (2018). One-step *in situ* detection of MiRNA-21 expression in single cancer cells based on biofunctionalized MoS_2_ nanosheets. ACS Appl. Mater. Interfaces 10, 350–360. 10.1021/acsami.7b18102 29239169

[B92] Ouyang J. Deng Y. Y. Chen W. S. Xu Q. F. Wang L. Q. Liu Z. J.. (2018). Marriage of artificial catalase and black phosphorous nanosheets for reinforced photodynamic antitumor therapy. J. Mater. Chem. B 6, 2057–2064. 10.1039/C8TB00371H 32254429

[B93] Qi C. Cai S. F. Wang X. H. Li J. Y. Lian Z. Sun S. S. (2016). Enhanced oxidase/peroxidase-like activities of aptamer conjugated MoS_2_/PtCu nanocomposites and their biosensing application. RSC Adv. 6, 54949–54955. 10.1039/C6RA03507H

[B94] Qian X. Gu Z. Chen Y. (2017). Two-dimensional black phosphorus nanosheets for theranostic nanomedicine. Mater. Horiz. 4, 800–816. 10.1039/C7MH00305F

[B95] Ren B. Y. Wang Y. C. Ou J. Z. (2020). Engineering two-dimensional metal oxides via surface functionalization for biological applications. J. Mater. Chem. B 8, 1108–1127. 10.1039/C9TB02423A 31971200

[B96] Ren X. Y. Huo M. F. Wang M. M. Lin H. Zhang X. X. Yin J.. (2019). Highly catalytic niobium carbide (MXene) promotes hematopoietic recovery after radiation by free radical scavenging. ACS Nano. 13, 6438–6454. 10.1021/acsnano.8b09327 31180624

[B97] Ruan Y. D. Jia X. D. Wang C. Zhen W. Y. Jiang X. E. (2018). Mn-Fe layered double hydroxide nanosheets: a new photothermal nanocarrier for O_2_-evolving phototherapy. Chem. Commun. 54, 11729–11732. 10.1039/C8CC06033A 30276370

[B98] Sasidharan A. Panchakarla L. Chandran P. Menon D. Nair S. Rao C.. (2011). Differential nano-bio interactions and toxicity effects of pristine versus functionalized graphene. Nanoscale 3, 2461–2464. 10.1039/c1nr10172b 21562671

[B99] Sasidharan A. Panchakarla L. S. Sadanandan A. R. Ashokan A. Chandran P. Girish C. M.. (2012). Hemocompatibility and macrophage response of pristine and functionalized graphene. Small 8, 1251–1263. 10.1002/smll.201102393 22334378

[B100] Shi Y. Hamsen C. Jia X. Kim K. K. Reina A. Hofmann M.. (2010). Synthesis of few- layer hexagonal boron nitride thin film by chemical vapor deposition. Nano Lett. 10, 4134–4139. 10.1021/nl1023707 20812716

[B101] Singh S. K. Singh M. K. Nayak M. K. Kumari S. Shrivastava S. Grácio J. J.. (2011). Thrombus inducing property of atomically thin graphene oxide sheets. ACS Nano. 5, 4987–4996. 10.1021/nn201092p 21574593

[B102] Song Y. J. Qu K. G. Zhao C. Ren J. S. Qu X. G. (2010). Graphene oxide: intrinsic peroxidase catalytic activity and its application to glucose detection. Adv. Mater. 22, 2206–2210. 10.1002/adma.200903783 20564257

[B103] Song Y. J. Wei W. L. Qu X. G. (2011). Colorimetric biosensing using smart materials. Adv. Mater. 23, 4215–4236. 10.1002/adma.201101853 21800383

[B104] Su L. Feng J. Zhou X. M. Ren C. L. Li H. H. Chen X. G. (2012). Colorimetric detection of urine glucose based ZnFe_2_O_4_ magnetic-nanoparticles. Anal. Chem. 84, 5753–5758. 10.1021/ac300939z 22702236

[B105] Sukumar T. Varghese J. Suja Bhargavan K. S. Jayasree P. Suvekbala V. Alaganandam K.. (2020). Cytotoxicity of formulated graphene and its natural rubber nanocomposite thin film in human vaginal epithelial cells: an influence of noncovalent interaction. ACS Biomater. Sci. Eng. 6, 2007–2019. 10.1021/acsbiomaterials.9b01897 32309635 PMC7157971

[B106] Sun H. Y. Liu X. L. Wang X. H. Han Q. S. Qi C. Li Y. M.. (2020). Colorimetric determination of ascorbic acid using a polyallylamine-stabilized IrO_2_/graphene oxide nanozyme as a peroxidase mimic. Microchim. Acta 187:110. 10.1007/s00604-019-3897-4 31916015

[B107] Sun X. M. Liu Z. Welsher K. Robinson J. T. Goodwin A. Zaric S.. (2008). Nano-graphene oxide for cellular imaging and drug delivery. Nano Res. 1, 203–212. 10.1007/s12274-008-8021-8 20216934 PMC2834318

[B108] Tan C. L. Cao X. H. Wu X. J. He Q. Y. Yang J. Zhang X.. (2017). Recent advances in ultrathin two-dimensional nanomaterials. Chem. Rev. 117, 6225–6331. 10.1021/acs.chemrev.6b00558 28306244

[B109] Tan C. L. Zhang H. (2015). Wet-chemical synthesis and applications of non-layer structured two-dimensional nanomaterials. Nat. Commun. 6:7873. 10.1038/ncomms8873 26303763 PMC4560752

[B110] Tang Y. Hu Y. Yang Y. X. Liu B. Y. Wu Y. G. (2020). A facile colorimetric sensor for ultrasensitive and selective detection of lead(II) in environmental and biological samples based on intrinsic peroxidase-mimic activity of WS_2_ nanosheets. Anal. Chim. Acta 1106, 115–125. 10.1016/j.aca.2020.01.043 32145839

[B111] Tao W. Kong N. Ji X. Y. Zhang Y. P. Sharma A. Ouyang J.. (2019). Emerging two-dimensional monoelemental materials (Xenes) for biomedical applications. Chem. Soc. Rev. 48, 2891–2912. 10.1039/C8CS00823J 31120049

[B112] Tao Y. Lin Y. H. Huang Z. Z. Ren J. S. Qu X. G. (2013). Incorporating graphene oxide and gold nanoclusters: a synergistic catalyst with surprisingly high peroxidase-like activity over a broad pH range and its application for cancer cell detection. Adv. Mater. 25, 2594–2599. 10.1002/adma.201204419 23418013

[B113] Tian J. Q. Liu Q. Asiri A. M. Qusti A. H. Al-Youbi A. O. Sun X. P. (2013). Ultrathin graphitic carbon nitride nanosheets: a novel peroxidase mimetic, Fe doping-mediated catalytic performance enhancement and application to rapid, highly sensitive optical detection of glucose. Nanoscale 5, 11604–11609. 10.1039/c3nr03693f 24121798

[B114] Vernekar A. A. Sinha D. Srivastava S. Paramasivam P. U. D'Silva P. Mugesh G. (2014). An antioxidant nanozyme that uncovers the cytoprotective potential of Vanadia nanowires. Nat. Commun. 5, 5301–5314. 10.1038/ncomms6301 25412933

[B115] Wang J. Y. Li W. Y. Zheng Y.-Q. (2019). Nitro-functionalized metal-organic frameworks with catalase mimic properties for glutathione detection. Analyst 144, 6041–6047. 10.1039/C9AN00813F 31508616

[B116] Wang K. Ruan J. Song H. Zhang J. Wo Y. Guo S. (2011). Biocompatibility of graphene oxide. Nanoscale Res. Lett. 6:8 10.1007/s11671-010-9751-6 27502632 PMC3212228

[B117] Wang S. G. Yang X. Q. Zhou L. L. Li J. F. Chen H. R. (2020). 2D nanostructures beyond graphene: preparation, biocompatibility and biodegradation behaviors. J. Mater. Chem. B 8, 2974–2989. 10.1039/C9TB02845E 32207478

[B118] Wang T. Zhang X. Mei L. Q. Ma D. Q. Liao Y. Zu Y.. (2020). A two-step gas/liquid strategy for the production of N-doped defect-rich transition metal dichalcogenide nanosheets and their antibacterial applications. Nanoscale 12, 8415–8424. 10.1039/D0NR00192A 32239043

[B119] Wang X. H. Han Q. S. Cai S. F. Wang T. Yang R. Wang C. (2017). Excellent peroxidase mimicking property of CuO/Pt nanocomposites and their application as an ascorbic acid sensor. Analyst 142, 2500–2506. 10.1039/C7AN00589J 28589198

[B120] Wang Y. Hu J. Zhuang Q. F. Ni Y. N. (2016). Enhancing sensitivity and selectivity in a label-free colorimetric sensor for detection of iron(II) ions with luminescent molybdenum disulfide nanosheet-based peroxidase mimetics. Biosens. Bioelectron. 80, 111–117. 10.1016/j.bios.2016.01.037 26807525

[B121] Wang Z. Z. Dong K. Liu Z. Zhang Y. Chen Z. W. Sun H. J. (2017). Activation of biologically relevant levels of reactive oxygen species by Au/*g*-C_3_N_4_ hybrid nanozyme for bacteria killing and wound disinfection. Biomaterials 113, 145–157. 10.1016/j.biomaterials.2016.10.041 27815998

[B122] Wei H. Wang E. K. (2013). Nanomaterials with enzyme-like characteristics (nanozymes): next-generation artificial enzymes. Chem. Rev. 42, 6060–6093. 10.1039/c3cs35486e 23740388

[B123] Wei J. P. Chen X. N. Shi S. G. Mo S. G. Zheng N. F. (2015). An investigation of the mimetic enzyme activity of two-dimensional Pd-based nanostructures. Nanoscale 7, 19018–19026. 10.1039/C5NR05675F 26515167

[B124] Wu N. Wang Y. T. Wang X. N. Guo F. N. Wen H. Yang T. (2019). Enhanced peroxidase-like activity of Au NPs loaded graphitic carbon nitride nanosheets for colorimetric biosensing. Anal. Chim. Acta 1091, 69–75. 10.1016/j.aca.2019.09.072 31679576

[B125] Wu X. J. Chen T. M. Wang J. X. Yang G. W. (2018). Few-layered MoSe_2_ nanosheets as an efficient peroxidase nanozyme for highly sensitive colorimetric detection of H_2_O_2_ and xanthine. J. Mater. Chem. B 6, 105–111. 10.1039/C7TB02434G 32254198

[B126] Wu Z. N. Liu J. L. Li Y. C. Cheng Z. Y. Li T. T. Zhang H.. (2015). Self-assembly of nanoclusters into mono-, few-, and multilayered sheets via dipole-induced asymmetric van der Waals attraction. ACS Nano. 9, 6315–6323. 10.1021/acsnano.5b01823 26030819

[B127] Xuan J. N. Wang Z. Q. Chen Y. Y. Liang D. J. Cheng L. Yang X. J.. (2016). Organic-based-driven intercalation and delamination for the production of functionalized titanium carbide nanosheets with superior photothermal therapeutic performance. Angew. Chem. Int. Ed. 55, 14569–14574. 10.1002/anie.201606643 27774723

[B128] Yan L. Gonca S. Zhu G. Y. Zhang W. J. Chen X. F. (2019). Layered double hydroxide nanostructures and nanocomposites for biomedical applications. J. Mater. Chem. B. 7, 5583–5601. 10.1039/C9TB01312A 31508652

[B129] Yan X. Song Y. Wu X. L. Zhu C. Z. Su X. G. Du D.. (2017). Oxidase-mimicking activity of ultrathin MnO_2_ nanosheets in colorimetric assay of acetylcholinesterase activity. Nanoscale 9, 2317–2323. 10.1039/C6NR08473G 28134376 PMC13068102

[B130] Yang C. Feng W. J. Li Y. Tian X. K. Zhou Z. X. Lu L. Q. (2019). A promising method for diabetes early diagnosis via sensitive detection of urine glucose by Fe-Pd/rGO. Dyes Pigments 164, 20–26. 10.1016/j.dyepig.2018.12.061

[B131] Yang H. Zhang X. Tang A. Qiu G. (2004). Gobalt ferrite nanoparticles prepared by coprecipitation/mechanochemical treatment. Chem. Lett. 33, 826–827. 10.1246/cl.2004.826

[B132] Yang S. B. Gong Y. J. Zhang J. S. Zhan L. Ma L. L. Fang Z. Y.. (2013). Exfoliated graphitic carbon nitride nanosheets as efficient catalysts for hydrogen evolution under visible light. Adv. Mater. 25, 2452–2456. 10.1002/adma.201204453 23450777

[B133] Yang W. N. Li J. Yang J. Liu Y. Xu Z. P. Sun X. F. (2020). Biomass-derived hierarchically porous CoFe-LDH/CeO_2_ hybrid with peroxidase-like activity for colorimetric sensing of H_2_O_2_ and glucose. J. Alloy Compound 815:152276 10.1016/j.jallcom.2019.152276

[B134] Yi M. Shen Z. (2015). A review on mechanical exfoliation for the scalable production of graphene. J. Mater. Chem. A 3, 11700–11715. 10.1039/C5TA00252D

[B135] Yim D. B. Lee D.-E. So Y. Choi C. Son W. Jang K. (2020). Sustainable nanosheet antioxidants for sepsis therapy via scavenging intracellular reactive oxygen and nitrogen species. ACS Nano. 14, 10324–10336. 10.1021/acsnano.0c03807 32806029

[B136] Yin W. Y. Yan L. Yu J. Tian G. Zhou L. J. Zheng X. P.. (2014). High-throughput synthesis of single-layer MoS_2_ nanosheets as a near-infrared photothermal-triggered drug delivery for effective cancer therapy. ACS Nano. 8, 6922–6933. 10.1021/nn501647j 24905027

[B137] Yong Y. Zhou L. J. Gu Z. J. Yan L. Tian G. Zheng X. P.. (2014). WS_2_ nanosheet as a new photosensitizer carrier for combined photodynamic and photothermal therapy of cancer cells. Nanoscale 6, 10394–10403. 10.1039/C4NR02453B 25047651

[B138] Yu F. T. Cangelosi V. M. Zastrow M. L. Tegoni M. Plegaria J. S. Tebo A. G. Mocny C. S.. (2014). Protein design: toward functional metalloenzymes. Chem. Rev. 114, 3495–3578. 10.1021/cr400458x 24661096 PMC4300145

[B139] Yuwen L. Yu H. Yang X. Zhou J. Zhang Q. Zhang Y.. (2016). Rapid preparation of single-layer transition metal dichalcogenide nanosheets via ultrasonication enhanced lithium intercalation. Chem. Commun. 52, 529–532. 10.1039/C5CC07301D 26535783

[B140] Zeng Z. Y. Yin Z. Y. Huang X. Li H. He Q. Y. Lu G.. (2011). Single-layer semiconducting nanosheets: high-yield preparation and device fabrication. Angew. Chem. Int. Ed. 50, 11093–11097. 10.1002/anie.201106004 22021163

[B141] Zhan T. R. Kang J. X. Li X. J. Pan L. Li G. J. Hou W. G. (2018). NiFe layered double hydroxide nanosheets as an efficiently mimic enzyme for colorimetric determination of glucose and H_2_O_2_. Sens. Actuat. B 255, 2635–2642. 10.1016/j.snb.2017.09.074

[B142] Zhang H. (2015). Ultrathin two-dimensional nanomaterials. ACS Nano. 9, 9451–9469. 10.1021/acsnano.5b05040 26407037

[B143] Zhang L. N. Deng H. H. Lin F. L. Xu X. W. Weng S. H. Liu A. L.. (2014). *In situ* growth of porous platinum nanoparticles on graphene oxide for colorimetric detection of cancer cells. Anal. Chem. 86, 2711–2718. 10.1021/ac404104j 24524671

[B144] Zhang T. Lu Y. Luo G. (2014). Synthesis of hierarchical iron hydrogen phosphate crystal as a robust peroxidase mimic for stable H_2_O2 detection. ACS Appl. Mater. Interfaces 6, 14433–14438. 10.1021/am503708a 25029358

[B145] Zhang W. C. Li X. Xu X. C. He Y. F. Qiu F. X. Pan J. M.. (2019). Pd nanoparticle-decorated graphitic C_3_N_4_ nanosheets with bifunctional peroxidase mimicking and ON-OFF fluorescence enable naked-eye and fluorescent dual-readout sensing of glucose. J. Mater. Chem. B 7, 233–239. 10.1039/C8TB02110D 32254548

[B146] Zhang X. L. Li G. L. Wu D. Li X. L. Hu N. Chen J.. (2019). Recent progress in the design fabrication of metal-organic frameworks-based nanozymes and their applications to sensing and cancer therapy. Biosens. Bioelectron. 137, 178–198. 10.1016/j.bios.2019.04.061 31100598

[B147] Zhang Y. Wang Y. N. Sun X. T. Chen L. Xu Z. R. (2017). Boron nitride nanosheet/CuS nanocomposites as mimetic peroxidase for sensitive colorimetric detection of cholesterol. Sens. Actuat. B 246, 118–126. 10.1016/j.snb.2017.02.059

[B148] Zhou X. F. Zheng X. L. Yan B. Xu T. Xu Q. (2017). Defect engineering of two-dimensional WO_3_ nanosheets for enhanced electrochromism and photoeletrochemical performance. Appl. Surf. Sci. 400, 57–63. 10.1016/j.apsusc.2016.12.072

[B149] Zhu C. F. Zeng Z. Y. Li H. Li F. Fan C. H. Zhang H. (2013). Single-layer MoS_2_-based nanoprobes for homogeneous detection of biomolecules. J. Am. Chem. Soc. 135, 5998–6001. 10.1021/ja4019572 23570230

